# Theacrine-rich Jianghua Kucha black tea alleviates depression *via* remodeling systemic tryptophan metabolism and targeting TPH1: insights from metabolomics and molecular simulation

**DOI:** 10.1039/d6ra00952b

**Published:** 2026-07-02

**Authors:** Xiaolu Yang, Guifen Wang, Chuanwei Zheng, Zhongjun Yan, Wei Liu, Aixiang Hou, Wenliang Wu

**Affiliations:** a College of Food Science and Technology, Hunan Agricultural University Changsha Hunan 410128 China aixianghou@163.com; b Hunan Institute of Tea Research, Hunan Academy of Agricultural Sciences Changsha Hunan 410125 China wwlvip8@163.com; c Longping Agricultural College, Hunan University Changsha Hunan 410082 China; d Chenzhou Institute of Agricultural Sciences Chenzhou Hunan 423000 China

## Abstract

Jianghua Kucha black tea (JH), a distinct variety characterized by its high theacrine content, exhibits significant antidepressant potential, however, bridging its systemic metabolic benefits to specific molecular targets remains a major challenge. In this study, we integrated untargeted serum metabolomics with multi-scale computational biology to explore the potential mechanism of action of JH in a chronic unpredictable mild stress (CUMS) mouse model. Rather than acting through a single pathway, metabolomic profiling revealed that JH induced a comprehensive reprogramming of the circulatory metabolic landscape. Specifically, JH attenuated the metabolic perturbations associated with the maladaptive shunting of tryptophan toward the kynurenine pathway, thereby favoring the restoration of serotonin (5-HT) biosynthesis precursors. To elucidate the molecular drivers behind this metabolic shift, integrative network pharmacology and atomistic molecular dynamics (MD) simulations were employed, prioritizing TPH1 as a high potential therapeutic target. The computational models predicted that JH's characteristic bioactives, notably theacrine and theaflavins, could form stable, high-affinity binding conformations with TPH1. Collectively, this study provides a novel “metabolism-target” structural framework, providing theoretical insights into the circulatory mechanisms of JH and highlighting its significant promise as a dietary intervention for mood disorders.

## Abstract 2

Jianghua Kucha black tea alleviates CUMS by regulating tryptophan metabolism to restore 5-HT, computationally predicting TPH1 as a potential target. Image created with the assistance of Google Gemini.

## Graphical abstract

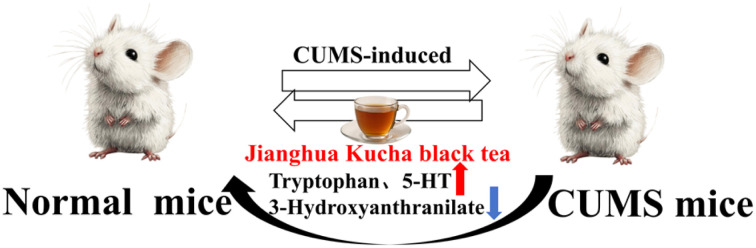

## 1. Introduction

Depression is a debilitating chronic mental disorder characterized by persistent low mood, anhedonia, and cognitive impairments, affecting approximately 5% of the adult population globally.^1^ Despite the availability of pharmacotherapies such as SSRIs, limitations such as delayed onset, drug resistance, and adverse effects necessitate the investigation of safer alternative strategies.^2^ Consequently, there is a growing interest in exploring natural functional foods that possess antidepressant properties with higher safety profiles, offering a promising complementary strategy for mood regulation.

Tea (*Camellia sinensis*), one of the most widely consumed non-alcoholic beverages globally, is rich in a variety of bioactive compounds. Accumulating evidence suggests that tea consumption is associated with a reduced risk of depression.^3–5^ Specific components, such as theanine and epigallocatechin gallate (EGCG), have been reported to modulate monoamine neurotransmitters like 5-HT and dopamine.^6–8^ Furthermore, theaflavins, the characteristic pigments in black tea, exhibit anti-neuroinflammatory effects and elevate brain dopamine, contributing to antidepressant outcomes.^9,10^ Notably, Jianghua Kucha, a unique tea germplasm, is distinguished by its high content of theacrine (1,3,7,9-tetramethyluric acid), a purine alkaloid, the theacrine has been demonstrated to possess antidepressant and sedative properties.^11–13^

Depression is increasingly recognized not merely as a localized brain disorder, but as a systemic physiological disruption intimately linked to peripheral metabolic dysregulation and inflammation. While conventional monoamine-targeted antidepressants remain the first-line clinical treatment, their adverse side effects and delayed efficacy have spurred a growing interest in nutritional psychiatry and dietary interventions. Natural functional beverages, particularly distinct tea varieties rich in unique purine alkaloids and polyphenols, offer a promising, multi-target alternative for mood management. However, a major bottleneck in the current field of nutritional psychiatry is the “black box” between systemic phenotypic improvements and exact molecular mechanisms. The bioactive components in functional foods are complex, making it extremely challenging to pinpoint which specific molecules bind to which exact protein targets to drive the observed circulatory metabolic corrections. Therefore, bridging the gap between macroscopic systemic metabolism and atomistic molecular interactions is of profound significance for validating the therapeutic rationale of dietary interventions.

In our recent investigation, we established that Jianghua Kucha black tea (JH) effectively mitigates depression-like behaviors in chronic unpredictable mild stress (CUMS) mice, potentially by modulating gut microbiota composition and function, as well as brain neurochemicals and cytokines.^14^ It is well established that the gut–brain axis relies on the circulatory system to transmit signals. Although our previous study revealed how JH modulates the gut microbiota, the critical link between intestinal changes and brain neuroprotection remains missing. Specifically, it remains unclear how JH alters systemic metabolism in the serum and which specific molecular targets are engaged by its characteristic bioactive components—whether through direct systemic absorption (*e.g.*, theacrine) or peripheral interaction (*e.g.*, theaflavins). Understanding these circulatory metabolic signatures and molecular interactions is valuable to further elucidate the antidepressant mechanism of JH.

To elucidate this complex “component-metabolism-target” network, an integrative strategy combining metabolomics and computational biology is essential. Serum metabolomics provides a holistic snapshot of the host's physiological state, capturing the dynamic metabolic flux resulting from both gut microbial processing and host absorption.^15^ Complementarily, network pharmacology and molecular dynamics (MD) simulations offer a powerful means to predict and validate ligand–protein interactions at the atomic level. Integrating these approaches allows us to identify core therapeutic targets and determine how distinct tea bioactives physically engage these targets to drive metabolic restoration.^16,17^

In this study, we employed a chronic unpredictable mild stress (CUMS) mouse model to investigate the systemic antidepressant mechanism of JH. Building upon the biological samples from our established cohort,^14^ we first performed metabolomics to map the metabolic changes in the blood. Based on the metabolomic findings, we hypothesized a core mechanism: JH may reduce the conversion of tryptophan into inflammatory byproducts (kynurenine pathway), thereby saving more tryptophan in the circulation to support brain 5-HT synthesis. To identify which proteins drive this metabolic shift, we used network pharmacology to screen for potential targets. Subsequently, performed 100 ns molecular dynamics (MD) simulations to evaluate the binding potential of JH active components, such as theacrine and theaflavins, could directly bind and stabilize this enzyme. The significance of this study lies in bridging the gap between macroscopic systemic metabolism and microscopic multi-component target engagement. Through this “Metabolite-Target” analysis, we not only provide a predictive mechanistic framework for JH's synergistic action, but also offer a solid scientific rationale for its application as a functional beverage in mood regulation.

## 2. Materials and methods

### 2.1. Tea preparation, and animal cohort

The Jianghua Kucha black tea used in this study was prepared, and its phytochemical profile was quantified as detailed in our previous publication.^14^ This study was conducted using biological samples (serum) and experimental data (behavioral and biochemical) derived from the same animal cohort detailed in our previous publication,^14^ thereby maximizing scientific data yield without additional animal sacrifice. Briefly, the experimental protocol was approved by the Animal Ethics Committee of the Hunan Academy of Agricultural Sciences (Approval No. SYXK (Xiang) 2020-0008). Male C57BL/6J mice were subjected to a 5-week chronic unpredictable mild stress (CUMS) procedure and treated with JH. The cohort included a Control (Con) group, a Model (Mod) group, a Fluoxetine (FLX) group, and CUMS groups treated with different doses of JH, JH low-dose (JH-L) and JH high-dose (JH-H).

For the current study, data regarding behavioral tests (sucrose preference test, open field test, and forced swimming test) and cerebral biochemical indices (including BDNF, 5-HT, DA and inflammatory cytokines) were retrieved from the original dataset to perform correlation analyses. The serum samples, which were collected at the time of sacrifice and stored at −80 °C, were thawed and processed specifically for the untargeted metabolomics analysis described below.

### 2.2. UPLC-Q-exactive-Orbitrap-MS/MS-based serum metabolomics

#### 2.2.1. Sample preparation

Serum samples were thawed on ice. A 100 µL aliquot of each sample was mixed with 400 µL of pre-chilled extraction solvent (methanol) to precipitate proteins. The mixture was vortexed, incubated on ice for 5 min, and centrifuged at 13 000×*g* for 15 min at 4 °C. The supernatant was filtered through a 0.22 µm membrane and transferred to LC-MS vials for analysis. To ensure data reliability, Quality Control (QC) samples were prepared by pooling equal volumes (10 µL) from each serum sample. These QC samples were inserted at regular intervals throughout the analytical run to monitor system stability.

#### 2.2.2. Instrumental analysis

Chromatographic separation was performed on a Thermo Fisher UHPLC system equipped with a Waters ACQUITY UPLC HSS T3 column (100 mm × 2.1 mm, 1.8 µm) maintained at 45 °C. The mobile phases consisted of 0.1% aqueous formic acid (A) and methanol (B) with a flow rate of 0.35 mL min^−1^. The gradient elution was: 0–1.5 min, 5% B; 1.5–3 min, 5–30% B; 3–5 min, 30–60% B; 5–7 min, 60–80% B; 7–12 min, 80–100% B; 12–16 min, 100% B; 16–16.5 min, 100–5% B. Mass spectrometric detection was carried out on a Q Exactive HF-X system operating in both positive and negative ion modes. Key parameters included: scan range, 60–900 *m*/*z*; full MS resolution, 70 000; MS/MS resolution, 17 500; spray voltages, 3.5 kV (positive) and 3.1 kV (negative); capillary temperature, 320 °C; and auxiliary gas heater temperature, 375 °C.

### 2.3. Network pharmacology analysis

#### 2.3.1. Target prediction of active compounds and collection of depression-related targets

Sixteen representative bioactive compounds were selected based on the quantitative chemical profile of JH extract (Table S2) and their reported antidepressant relevance.^6,8,9,11^ These compounds were categorized into purine alkaloids (*e.g.*, theacrine, caffeine), amino acids (theanine), tea polyphenols (*e.g.*, catechins) and catechin oxidation polymers (*e.g.*, theaflavins). The 2D structures were retrieved from PubChem (https://pubchem.ncbi.nlm.nih.gov/). Potential targets were predicted using PharmMapper (https://www.lilab-ecust.cn/pharmmapper/), SwissTargetPrediction (https://www.swisstargetprediction.ch/), and SEA Search Server (https://sea.bkslab.org/) based on isomeric SMILES. Target names were standardized to official gene symbols using the UniProt database (https://www.uniprot.org/), limiting the species to “*Homo sapiens*” to ensure maximum database coverage. Depression-related targets were collated from GeneCards (https://www.genecards.org/) (relevance score >1), DisGeNET (https://www.disgenet.org/) (score >0.1), TTD (https://db.idrblab.net/ttd/), and OMIM (https://omim.org/) databases. The intersection of JH-predicted targets and disease targets was visualized using a Venn diagram.

#### 2.3.2. Network construction and enrichment analysis

A Compound-Target-Disease (C-T-D) network was constructed using Cytoscape 3.10.2. A Protein–Protein Interaction (PPI) network was built *via* the STRING database (https://string-db.org/) (confidence score >0.4). Gene Ontology (GO) and KEGG pathway enrichment analyses were performed using the DAVID platform (https://davidbioinformatics.nih.gov/) to elucidate biological mechanisms.

### 2.4. Molecular docking and molecular dynamics (MD) simulations

#### 2.4.1. Molecular docking

Molecular docking was performed using AutoDock Vina 1.5.7 following described protocols.^18^ Briefly, the 2D structures of the active compounds were obtained from the PubChem database (https://pubchem.ncbi.nlm.nih.gov/), and converted to 3D structures using Chem3D software (PerkinElmer Informatics, Waltham, MA, USA). These structures were then energy-minimized and exported in mol2 format. Protein conformations of key targets, specifically TPH1(PDB:1MLW), along with CYP2C9, CYP1A2, CAT, and ACHE, were retrieved from the Protein Data Bank (PDB, https://www.rcsb.org/) and processed using PyMol software. The ligand and protein receptor molecules were prepared using AutodockTools 1.5.7. The results were visualized and analyzed using Discover Studio 2021 software (Dassault Systèmes, San Diego, CA, USA).

#### 2.4.2. Molecular dynamics (MD) simulations

To rigorously validate the stability of the ligand–protein complexes identified *via* docking, 100 ns MD simulations were performed using GROMACS 2023.5. The protein topology was prepared using the AMBER99SB-ILDN force field, while ligand topologies were generated using ACPYPE. The systems were solvated in a cubic box (1.0 nm margin) with the TIP3P water model and neutralized with Na^+^ and Cl^−^ ions. Following steepest descent energy minimization, the systems underwent 100 ps of equilibration in both NVT and NPT ensembles. Production runs were executed at 300 K (V-rescale thermostat) and 1 bar (Parrinello-Rahman barostat). Trajectory stability was evaluated *via* Root Mean Square Deviation (RMSD), Root Mean Square Fluctuation (RMSF), Radius of Gyration (*R*_g_), and Solvent Accessible Surface Area (SASA). Finally, the binding free energy was calculated using the Molecular Mechanics/Poisson Boltzmann Surface Area (MM/PBSA) method based on the stable trajectory of the last 20 ns.^19^

### 2.5. Statistical and data analysis

Data are expressed as mean ± standard deviation (SD). Multivariate statistical analyses (PCA, OPLS-DA) were performed using SIMCA 14.1 (Umetrics, Umeå, Sweden). Heatmaps were generated *via* MultiExperiment Viewer 4.9.0 (Oracle, Redwood, CA, USA). The correlation heatmap and functional enrichment visualizations were performed using the Metware Cloud platform (https://cloud.metware.cn/). Network diagrams were using Cytoscape 3.10.2 software. Spearman's correlation analysis was conducted using SPSS 25.0 (IBM Corp., Armonk, NY, USA) to evaluate the associations between differential serum metabolites identified in this study and the cerebral biochemical/inflammatory indices obtained from the preceding study. Statistical significance was defined as *P < 0.05 and **P < 0.01.

## 3. Results

### 3.1. Confirmation of the pharmacodynamic efficacy of JH (summary of the established cohort)

As the fundamental prerequisite for the current omics and computational analyses, the *in vivo* antidepressant pharmacodynamics of Jianghua Kucha black tea (JH) were thoroughly established in the initial phase of this project using the same animal cohort.^14^ Briefly, compared to the Control group, the CUMS procedure successfully induced core depression-like phenotypes, whereas continuous oral administration of JH effectively reversed these behavioral deficits. Furthermore, biochemical analyses from that cohort confirmed that JH intervention significantly attenuated systemic inflammation (*e.g.*, IL-6, TNF-α) and restored brain monoamine neurotransmitters (*e.g.*, 5-HT, DA) levels.^14^ Building upon this confirmed efficacy, the present study utilized the biobanked serum samples from this exact cohort to decipher the underlying systemic metabolic reprogramming and precise molecular targets.

### 3.2. JH reshaped the serum metabolic landscape in CUMS mice

Building upon the antidepressant efficacy of JH observed in our previous cohort.^14^ the current study employed serum metabolomics to map the circulatory metabolic profile and identify potential therapeutic targets associated with these benefits. To achieve this, UPLC-Q-Exactive-Orbitrap-MS/MS untargeted metabolomics was performed on serum samples.

**Fig. 1:**
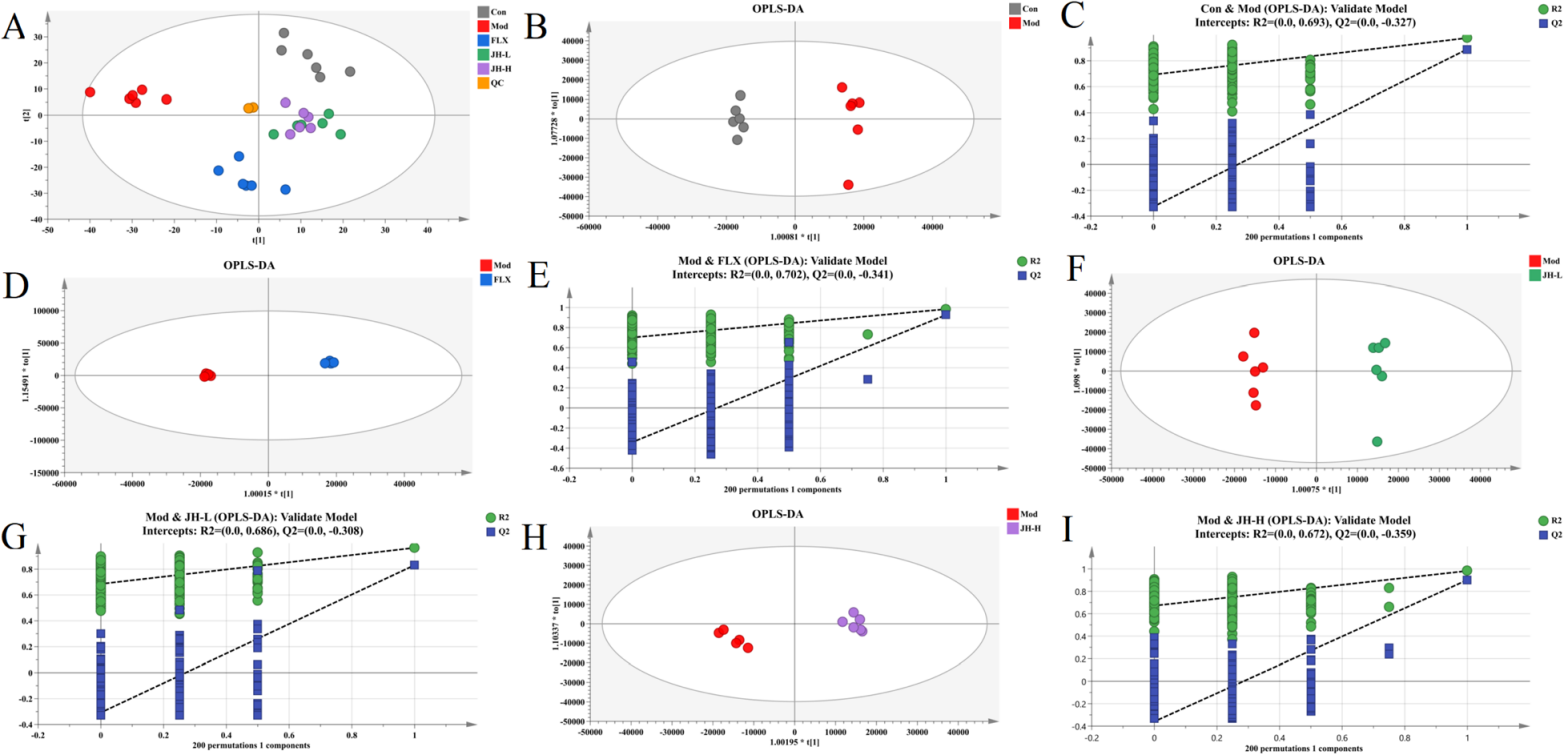
Multivariate statistical analysis of serum metabolomics profiles in CUMS mice. (A) PCA score plots demonstrating the separation among five groups; (B, D, F and H) OPLS-DA score plots comparing Con *vs.* Mod, Mod *vs.* FLX, Mod *vs.* JH-L, and Mod *vs.* JH-H, respectively. (C, E, G and I) permutation tests (*n* = 200) validating the OPLS-DA models. Note: data are presented as mean ± SD (*n* = 6).

First, Principal Component Analysis (PCA) was employed to visualize the intrinsic metabolic patterns. As shown in Fig. 1A, the Quality Control (QC) samples clustered tightly, validating the system's stability. A distinct separation was observed between the Control (Con) and Model (Mod) groups, confirming that chronic stress induced profound perturbations in the host circulatory metabolome. Notably, the metabolic trajectories of JH-treated mice (both JH-L and JH-H) diverged from the Mod group and shifted towards the Con group, indicating an overall amelioration of the stress-induced metabolic profiles following JH intervention.

To identify potential biomarkers driving these shifts, supervised Orthogonal Partial Least Squares Discriminant Analysis (OPLS-DA) was performed. The score plots revealed clear segregations for all pairwise comparisons (Mod *vs.* Con/FLX/JH-L/JH-H) (Fig. 1B, D, F, H). The robustness of these models was rigorously confirmed by 200-iteration permutation tests, where all permuted *R*^2^ and *Q*^2^ values were lower than the original ones (Fig. 1C, E, G and I), ruling out overfitting.

**Fig. 2:**
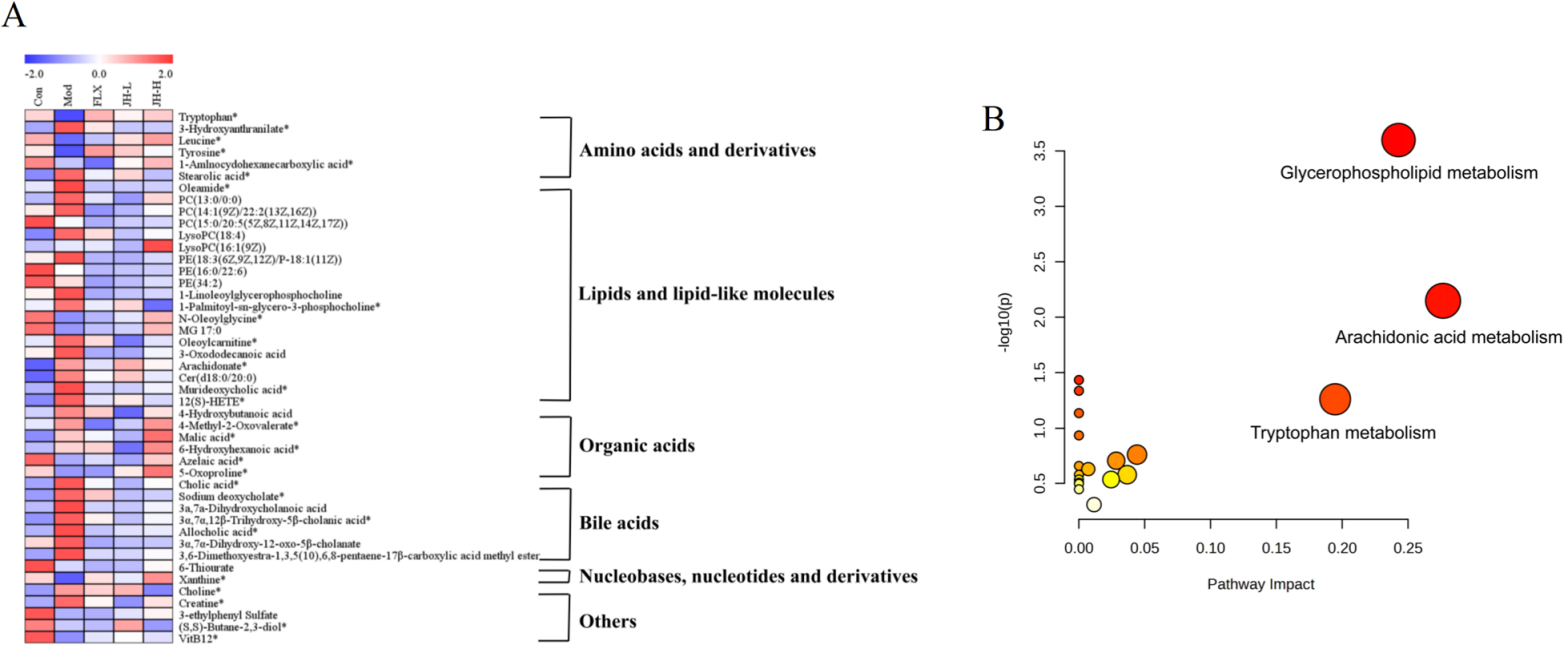
Visualization of differential serum metabolites and metabolic pathway analysis. (A) Heatmap of differential metabolites contents in different groups. The color scale represents the normalized intensity (red: upregulation; blue: downregulation); (B) metabolic pathway analysis revealing the key pathways disturbed by stress and regulated by JH. Note: an asterisk (*) indicates compounds validated by authentic standards. Data are presented as mean ± SD (*n* = 6).

Based on the criteria of VIP > 1 and *P* < 0.05, 45 differential metabolites were identified (Table 1). Hierarchical clustering analysis (Heatmap, Fig. 2A) visualized distinct metabolic signatures. These metabolites spanned multiple chemical classes, primarily amino acids and derivatives (5), lipids and lipid-like molecules (20), organic acids (6), bile acids (7), nucleobases, nucleotides and derivatives (2) and others, specifically, CUMS mice exhibited elevated levels of pro-inflammatory and stress-related markers (*e.g.*, 3-hydroxyanthranilate, arachidonate, 12(*S*)-HETE, and cholic acid) alongside depleted levels of essential neurotransmitter precursors (*e.g.*, tryptophan and tyrosine). Crucially, JH intervention exerted a significant restorative trend on the circulatory metabolome: it statistically reversed the levels of 34 out of 45 stress-induced metabolites, realigning their expression profiles towards the normal control. While metabolomic profiling primarily reveals associations, the normalization of these specific key metabolites, particularly the preservation of neurotransmitter precursors such as tryptophan and the suppression of pro-inflammatory mediators, provides a critical biochemical rationale for JH's antidepressant efficacy, which guided our subsequent pathway and target network analyses.

**Table 1:** Identification of potential serum biomarkers in CUMS mice treated with JH^a^

No.	RT	*m*/*z*	Metabolite	Main MS/MS fragments	Ion mode	Change trend			
						Mod/Con	FLX/Mod	JH-L/Mod	JH-H/Mod
1	4.63	205.0971	Tryptophan*	188.0708, 170.0605, 146.0602, 118.0605, 105.0037	ESI+	↓^##^	↑^##^	↑^##^	↑^##^
2	1.76	132.1020	Leucine*	109.2576, 86.0970, 69.0706	ESI+	↓^##^	↑^#^	↑^##^	↑^##^
3	1.57	182.0812	Tyrosine*	165.0548, 136.0759, 123.0443, 91.0547, 65.0391	ESI+	↓^##^	↑^##^	↑^##^	↑^##^
4	0.83	144.1018	1-Amlnocydohexanecarboxylic acid*	129.0785, 98.0604, 58.0659	ESI+	↓^##^	↑^##^	↑	↑^##^
5	4.89	154.0418	3-Hydroxyanthranilate*	119.0732, 91.0547, 65.0393	ESI+	↑^##^	↓^#^	↓^##^	↓^##^
6	12.28	279.2331	Stearolic acid*	170.7610, 112.5902, 79.3600	ESI−	↑^#^	↓	↓	↓^#^
7	12.00	282.2790	Oleamide*	247.2423, 134.0966, 97.1016, 83.0861, 69.0706	ESI+	↑^##^	↓^##^	↓^##^	↓^##^
8	8.13	514.2846	LysoPC(18:4)	124.0063,79.9562	ESI−	↑^##^	↓^#^	↓^##^	↓^##^
9	10.73	520.3397	1-Linoleoylglycerophosphocholine	184.0735, 125.0000, 86.0970, 60.0815	ESI+	↑^##^	↓^##^	↓^##^	↓^##^
10	11.03	496.3390	1-Palmitoyl-*sn*-glycero-3-phosphocholine*	184.0735, 125.0000, 86.0970, 60.0815	ESI+	↑^#^	↓^#^	↓	↓^##^
11	8.07	340.2819	*N*-Oleoylglycine*	241.5271, 106.1271, 57.4556	ESI+	↓^##^	↑	↑^#^	↑^##^
12	15.54	784.5849	PC (14:1(9Z)/22:2(13*Z*,16*Z*))	184.0735, 125.0000, 86.0970	ESI+	↑^##^	↓^##^	↓^##^	↓^##^
13	8.10	362.3262	MG 17:0	300.2899, 256.2632, 132.1021, 70.0658	ESI+	↓^##^	↑	↑	↑^##^
14	9.73	426.3571	Oleoylcarnitine*	367.2840, 297.2071, 144.1021, 85.0289, 60.0815	ESI+	↑^#^	↓	↓^##^	↓^#^
15	8.85	213.1492	3-Oxododecanoic acid	213.1488, 144.9116, 87.7787	ESI−	↑^##^	↓^##^	↓^##^	↓^##^
16	10.42	304.2348	Arachidonate*	280.5719, 222.9333, 157.5387, 101.9176	ESI+	↑^##^	↓^#^	↓	↓
17	12.94	596.5964	Cer(d18:0/20:0)	340.3571, 311.2943, 283.2633, 102.0917, 57.0706	ESI+	↑^##^	↓^##^	↓	↓^##^
18	15.53	722.5129	PE (18:3(6Z,9Z,12Z)/P-18:1(11*Z*))	436.2818, 303.2329, 140.0113, 78.9578	ESI−	↑^##^	↓^##^	↓^##^	↓^##^
19	9.43	391.2857	Murideoxycholic acid*	345.2796, 272.3132, 138.6871, 91.3073	ESI−	↑^##^	↓^##^	↓^##^	↓^##^
20	8.13	512.2690	PC (13:0/0:0)	184.0735, 125.0001, 104.1074, 86.0970, 60.0815	ESI+	↑^##^	↓^##^	↓^##^	↓^##^
21	10.44	320.2309	12(*S*)-HETE*	319.2277, 115.9196	ESI−	↑^##^	↓^#^	↓	↓^#^
22	1.80	103.0390	4-Hydroxybutanoic acid	103.0390, 59.0127	ESI−	↑^#^	↓	↓^##^	↓
23	0.94	130.0501	5-Oxoproline*	102.0552, 84.0813	ESI+	↓^##^	↑	↑^##^	↑^##^
24	9.58	431.2768	Cholic acid*	255.1680, 226.9513, 158.9632, 90.9771, 62.9824	ESI+	↑^##^	↓^#^	↓^##^	↓^#^
25	10.48	415.2817	Sodium deoxycholate*	383.1896, 119.0858, 56.9656	ESI+	↑^##^	↓	↓^##^	↓^##^
26	10.51	391.2857	3a,7a-Dihydroxycholanoic acid	345.2792, 255.2324	ESI−	↑^##^	↓^##^	↓^##^	↓^##^
27	9.60	407.2803	3α,7α,12β-Trihydroxy-5β-cholanic acid*	389.2692, 343.2642, 289.2174	ESI−	↑^##^	↓^##^	↓^##^	↓^##^
28	8.79	407.2800	Allocholic acid*	371.2575, 259.3816	ESI−	↑^##^	↓^##^	↓^##^	↓^##^
29	8.70	405.2642	3α,7α-Dihydroxy-12-oxo-5β-cholanate	343.2635, 289.2128, 123.0804	ESI−	↑^##^	↓^##^	↓^##^	↓^##^
30	8.35	355.1882	3,6-Dimethoxyestra-1,3,5(10),6,8-pentaene-17β-carboxylic acid methyl ester	285.0098, 105.0878, 91.0577, 73.0474	ESI+	↑^##^	↓^##^	↓^##^	↓^##^
31	1.50	153.0408	Xanthine*	128.0456, 110.0352, 97.0652	ESI+	↓^##^	↑^##^	↑^##^	↑^##^
32	0.74	104.1073	Choline*	86.0968, 60.0815	ESI+	↑^##^	↓	↓	↓^#^
33	0.79	132.0768	Creatine*	114.0666, 90.0555, 68.0502	ESI+	↑^##^	↓^##^	↓^##^	↓^#^
34	14.05	301.1408	VitB12*	240.0995, 184.0730, 104.1074, 81.1836	ESI+	↓^##^	↑^##^	↑^##^	↑

a The levels of potential biomarkers were labeled with up-regulation (↑) and down-regulation (↓). #: *P* < 0.05, ##: *P* < 0.01. An asterisk (*) indicates compounds validated by authentic standards. Data are presented as mean ± SD (*n* = 6).

Pathway enrichment analysis (MetaboAnalyst 6.0) further elucidated the biological impact of these alterations. As depicted in Fig. 2B and Table S1, 19 metabolic pathways were implicated. Based on pathway impact (>0.15) and significance (−log_10_(*p*) > 1.3), glycerophospholipid metabolism, arachidonic acid metabolism, and tryptophan metabolism emerged as the most critical pathways disrupted by chronic stress and effectively regulated by JH treatment.

### 3.3. Correlation analysis between serum metabolites and neuro-behavioral phenotypes

**Fig. 3:**
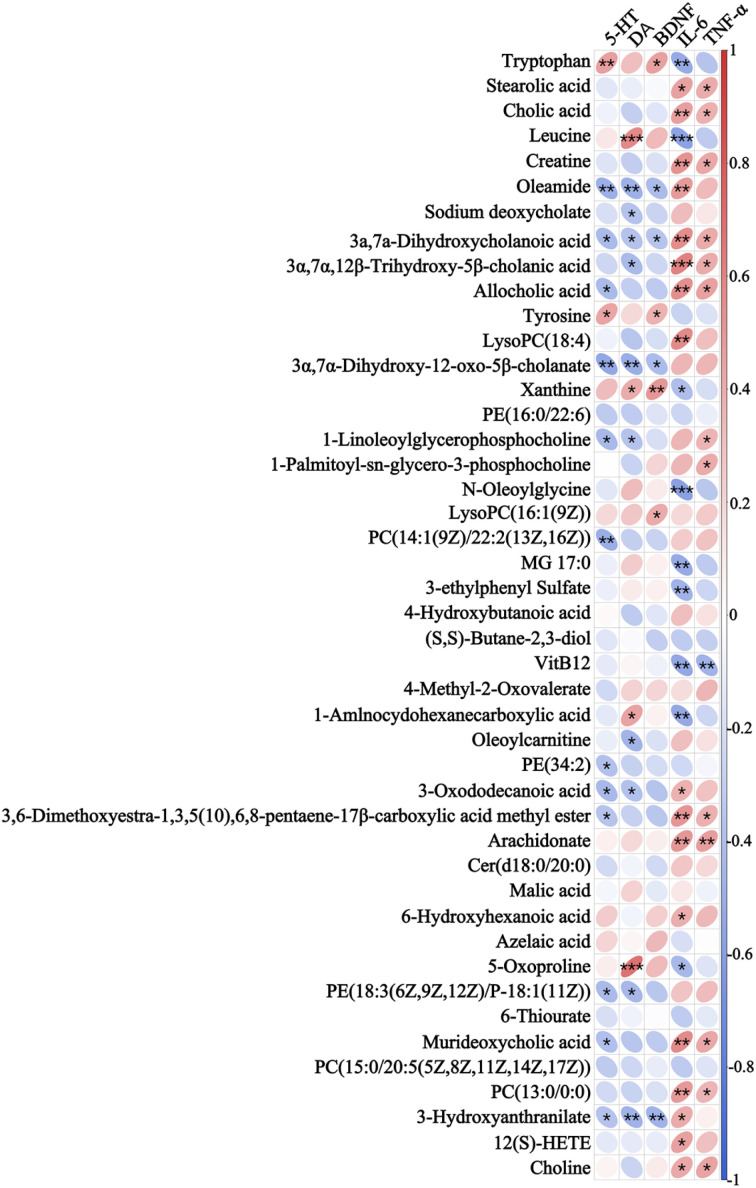
Correlation analysis between differential serum metabolites and brain biochemical indices (neurotransmitters and inflammatory cytokines). The correlation heatmap illustrates the Spearman correlation coefficients (*r*), with red indicating positive correlations and blue indicating negative correlations. Color intensity is proportional to the magnitude of the correlation. Significant correlations are marked with asterisks (*p < 0.05, **p < 0.01, ***p < 0.001). Data for brain biochemical indices were retrieved from our previous study.^14^ Data are presented as mean ± SD (*n* = 6).

To bridge the gap between peripheral metabolic signatures and central depressive behaviors, we performed a Spearman correlation analysis integrating the identified serum biomarkers with the cerebral indices (BDNF, 5-HT, DA) and inflammatory cytokines (IL-6, TNF-α) indices obtained from our established *in vivo* cohort.^14^ (Fig. 3). The resulting correlation matrix revealed two distinct functional clusters:

(1) The tryptophan–serotonin axis: serum tryptophan levels exhibited robust positive correlations with brain 5-HT (*p* < 0.01) and BDNF (*p* < 0.05), while showing a strong inverse correlation with the pro-inflammatory cytokine IL-6 (*p* < 0.01). Conversely, 3-hydroxyanthranilate (3-HAA), a downstream metabolite of the kynurenine pathway, displayed an opposite pattern: negative associations with neurotrophic factors and positive associations with inflammatory markers. This dichotomy statistically supports the “inflammation-driven tryptophan shunt” hypothesis, where stress-induced inflammation diverts tryptophan away from 5-HT synthesis.

(2) The lipid–inflammation axis: pro-inflammatory lipid mediators, specifically arachidonate and 12(*S*)-HETE, were positively correlated with systemic inflammatory markers (IL-6, TNF-α).

Collectively, these data establish a “Metabolism–Inflammation–Brain” regulatory network. The mitigation of systemic inflammation is explicitly supported by our integrated findings: our preceding biochemical analysis demonstrated that JH suppressed the CUMS-induced elevation of central pro-inflammatory cytokines (IL-6 and TNF-α),^14^ while the current metabolomics revealed a concurrent downregulation of circulating pro-inflammatory lipid mediators (arachidonate and 12(*S*)-HETE) and the inflammation-driven metabolite 3-HAA. The strong positive correlations between these peripheral and central markers (Fig. 3) provide solid evidence that JH effectively dampens the systemic inflammatory response. By mitigating systemic inflammation, JH curtails the pathological shunting of tryptophan toward the kynurenine pathway, thereby preserving circulating tryptophan pools for central neurotransmitter synthesis and exerting its antidepressant effects.

### 3.4. Network pharmacology unveils the multi-target mechanism of JH

**Fig. 4:**
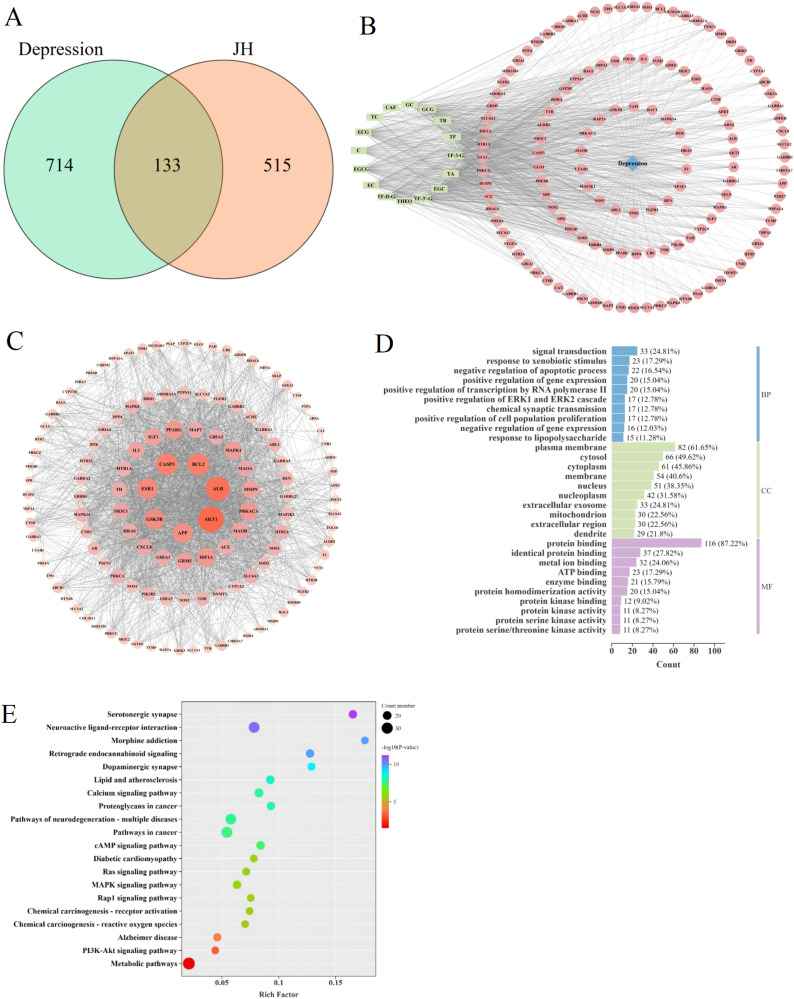
Network pharmacology analysis predicting the anti-depressive mechanism of JH. (A) Venn diagram showing the intersection targets between JH active components and depression-related targets; (B) compound-target-disease (C-T-D) network; (C) protein–protein interaction (PPI) network of potential targets; larger nodes and darker colors indicate higher degree centrality; (D) histogram of GO enrichment analysis involving biological processes (BPs), cell components (CCs), and molecular functions (MFs); (E) bubble chart of the top enriched KEGG pathways.

To elucidate the molecular initiators, we first identified potential targets for 16 key bioactive compounds of JH (including theacrine, theaflavins, and catechins) and mined depression-associated targets from public databases. The intersection of these two datasets yielded 133 potential therapeutic targets (Fig. 4A), which served as the foundation for subsequent mechanistic exploration.

To systematically visualize the interactions, a “Compound-Target-Disease” network was constructed (Fig. 4B), comprising 150 nodes and 1135 edges. The mapping between active components and putative targets was determined by molecular structural similarity and pharmacophore matching algorithms (*via* SwissTargetPrediction, PharmMapper and SEA Search Server). Furthermore, their links to “depression” were strictly filtered using specific database thresholds (*e.g.*, GeneCards relevance score > 1, DisGeNET score > 0.1) to ensure robust biological relevance. Topological analysis (Table S3) revealed that epicatechin gallate (ECG), theanine (TA), epigallocatechin gallate (EGCG) ranked highest in degree values, followed by the characteristic theaflavins (TF-3′-G, TF-D-G, TF-3-G) (degree > 80). The signature alkaloid theacrine (TC), despite having a lower degree (30) potentially due to fewer database annotations, was identified as a key node connecting multiple depression-related targets. Given its unique presence and stability in Jianghua Kucha, it was retained as a core component for subsequent analysis along with catechins and theaflavins.

Furthermore, the Protein–Protein Interaction (PPI) network (Fig. 4C) revealed a complex interactome consisting of 130 nodes and 2342 edges. The interactions between these targets were sourced from the STRING database. Here, the edges were established based on a stringent confidence score threshold (>0.400). Based on topological degree centrality, key hub targets with the highest connectivity included Albumin (ALB), Caspase-3 (CASP3), B-cell lymphoma-2 (BCL2), Estrogen Receptor 1 (ESR1), Amyloid Beta Precursor Protein (APP), AKT Serine/Threonine Kinase 1 (AKT1), and Glycogen Synthase Kinase 3 Beta (GSK3B). The high centrality of these genes suggests that JH may exert its effects by modulating critical neuroprotective, apoptotic, and synaptic plasticity pathways.

Further functional insights were gained through enrichment analyses. Gene Ontology (GO) analysis (Fig. 4D) indicated that these targets are primarily located in the synaptic parts and involve neurotransmitter receptor binding. KEGG pathway analysis (Fig. 4E and Table S4) was evaluated using both the enrichment significance (*p* < 0.05) and the gene count (number of targets enriched in the pathway). Crucially, their functional cross-validation with our upstream metabolomics findings. Among the top 20 pathways, the “serotonergic synapse” and “neuroactive ligand–receptor interaction” were highly significant. This finding aligns perfectly with the “tryptophan–serotonin” metabolic axis revealed by our serum metabolomics analysis. Additionally, pathways related to lipid metabolism were also enriched, corroborating the observed disturbances in glycerophospholipid and arachidonic acid metabolism. These results collectively suggest that JH restores homeostasis by simultaneously modulating synaptic transmission and metabolic flux.

### 3.5. Integrated analysis identifying TPH1 as a potential core mechanistic target

**Fig. 5:**
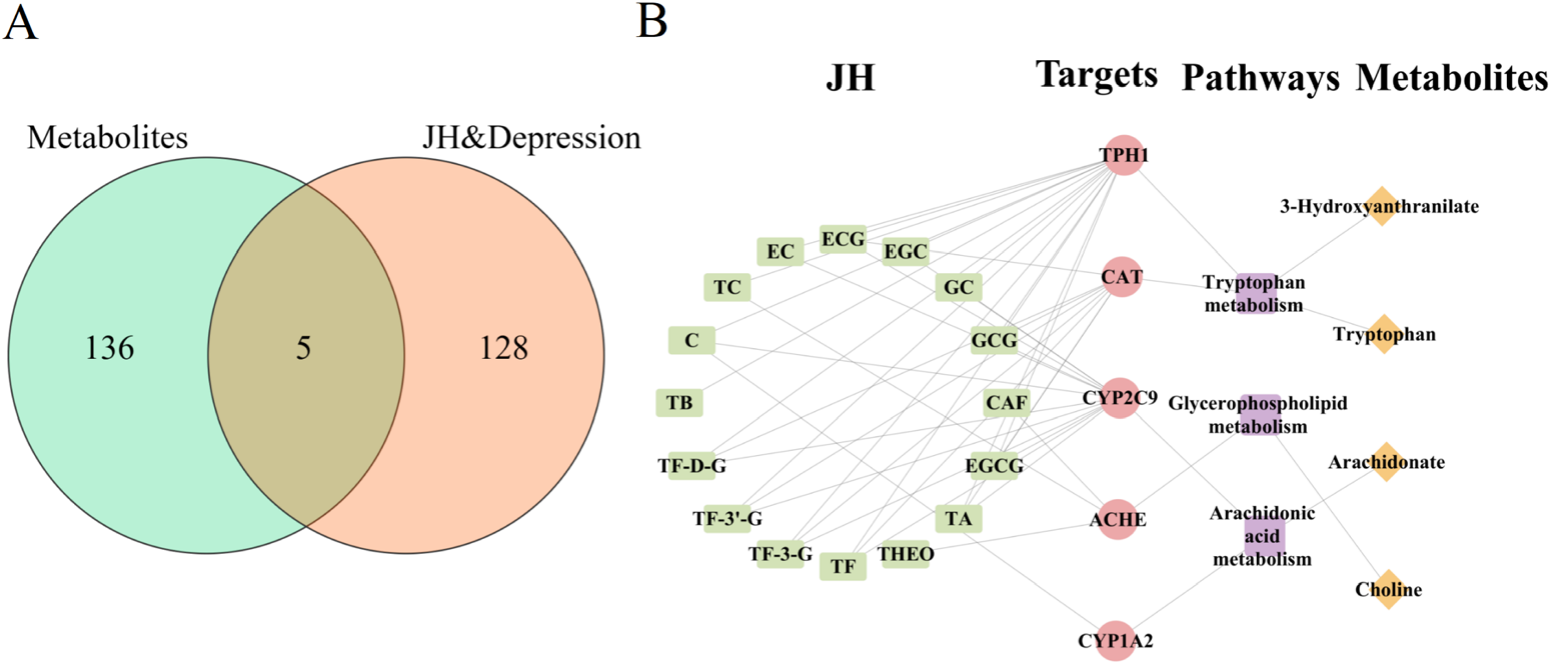
Integrated analysis of serum metabolomics and network pharmacology. (A) Venn diagram identifying the overlapping targets between metabolic pathway-related enzymes and JH-regulated targets; (B) the interaction network of key active components of JH-targets–metabolic pathways–metabolites.

To dissect the molecular drivers linking metabolic phenotypes to therapeutic targets, we employed an “Omics-Intersection” strategy. First, the 34 differential metabolites reversed by JH were imported into Cytoscape, where the MetScape plugin was utilized to construct a “Metabolite–Reaction–Enzyme–Gene” network (Fig. S1). This step predicted 141 potential upstream targets directly regulated by these metabolites. Subsequently, a Venn analysis (Fig. 5A) was conducted to intersect these metabolite-derived targets with the 133 JH-derived targets. This strategy narrowed down the candidates to 5 core targets: Cytochrome P450 2C9 (CYP2C9), Cytochrome P450 1A2 (CYP1A2), Tryptophan Hydroxylase 1 (TPH1), Catalase (CAT), and Acetylcholinesterase (ACHE).

To visualize the connectivity, a comprehensive interaction network was constructed (Fig. 5B). This network revealed that these core targets primarily regulate tryptophan metabolism, glycerophospholipid metabolism, and arachidonic acid metabolism, closely associating with key metabolites such as 3-hydroxyanthranilate, tryptophan, arachidonate, and choline.

Notably, TPH1 emerged as the most critical node in this regulatory map based on both topological ranking and biological relevance. Topologically, among the five candidate core targets in the comprehensive network (Table S3), TPH1 exhibited the highest degree centrality (degree = 15), followed by CYP2C9 (13), CAT (9), ACHE (4), and CYP1A2 (2), indicating its superior connectivity with JH bioactives and depression phenotypes. Functionally, KEGG enrichment (Table S4) revealed that TPH1 serves as a unique bridge connecting peripheral metabolic pathways (“tryptophan metabolism”) with central neural signaling (“serotonergic synapse”). Biologically, TPH1 is the rate-limiting enzyme for the biosynthesis of peripheral serotonin (5-HT). Given that our metabolomics data demonstrated a specific restoration of the “tryptophan–serotonin” axis, TPH1 was robustly prioritized as the most mechanistically relevant target for subsequent structural validation.

### 3.6. Molecular docking suggests potential high-affinity binding

To validate the predicted interactions, molecular docking was performed to assess the binding affinity between JH active components and the five core targets (Table 2). Generally, binding energies lower than −5.0 kcal mol^−1^ and −7.0 kcal mol^−1^ indicate good and strong binding activities, respectively.^20^ Strikingly, theaflavin derivatives (TF, TF-3-G, TF-3′-G, and TF-D-G) displayed the most potent binding potentials (energies ranging from −8.5 to −13.9 kcal mol^−1^), consistently outperforming the reference drug Fluoxetine (FLX). This suggests that these catechin oxidation polymers (theaflavins) in black tea may serve as high-affinity ligands for these metabolic enzymes.

**Table 2:** Binding energies of active components in JH and core targets (kcal mol^−1^)

	CYP2C9 PDB:1R9O	CYP1A2 PDB:2HI4	TPH1 PDB:1MLW	CAT PDB:8HID	ACHE PDB:4 M0E
Theacrine (TC)	−6.4	−7.7	−6.9	−8.2	−6.3
Caffeine (CAF)	−6.0	−7.3	−6.6	−7.9	−6.0
Theobromine (TB)	−5.6	−6.6	−6.1	−7.3	−6.9
Theophylline (THEO)	−6.0	−6.9	−6.3	−7.4	−5.1
Theanine (TA)	−5.1	−5.9	−6.2	−6.2	−6.1
Catechin (C)	−7.8	−9.4	−10.1	−10.4	−9.0
Epicatechin (EC)	−7.8	−9.1	−9.5	−9.9	−8.3
Epicatechin gallate (ECG)	−8.9	−10.4	−11.1	−8.9	−9.8
Epigallocatechin (EGC)	−8.2	−8.7	−9.4	−8.4	−8.2
Epigallocatechin gallate (EGCG)	−8.4	−10.1	−10.5	−10.5	−9.7
Gallocatechin (GC)	−8.6	−8.6	−10.0	−9.9	−8.6
Gallocatechin gallate (GCG)	−10.2	−10.7	−10.5	−10.2	−9.9
Theaflavin (TF)	−9.5	−9.1	−12.7	−11.3	−10.1
Theaflavin 3-gallate (TF-3-G)	−10.0	−10.1	−12.3	−11.6	−10.6
Theaflavin 3′-gallate (TF-3′-G)	−9.5	−9.4	−12.4	−12.7	−10.2
Theaflavin-3,3′-digallate (TF-D-G)	−8.6	−8.5	−11.3	−13.9	−10.7
Fluoxetine (FLX)	−8.0	−9.5	−9.6	−9.2	−8.0

**Fig. 6:**
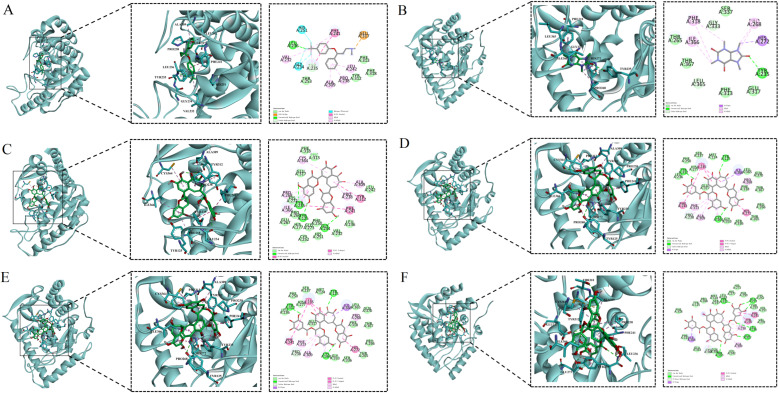
Molecular docking interactions between key active components of JH and the core target TPH1 (tryptophan hydroxylase 1). The 2D and 3D binding modes are shown for: (A) FLX (positive control, −9.6 kcal mol^−1^); (B) TC (−6.9 kcal mol^−1^); (C) TF (−12.7 kcal mol^−1^); (D) TF-3-G (−12.3 kcal mol^−1^); (E) TF-3′-G (−12.4 kcal mol^−1^); and (F) TF-D-G (−11.3 kcal mol^−1^).

Focusing on the priority target TPH1 (Fig. 6 and Table 2), docking simulations revealed that both TC and theaflavins fit snugly into the catalytic pocket. TF-3′-G achieved a remarkable binding energy of −12.4 kcal mol^−1^, significantly superior to FLX (−9.6 kcal mol^−1^). Visualization of the docking complexes (Fig. 6 and S2) highlighted distinct binding modes: theaflavins and FLX were anchored by a comprehensive network involving hydrogen bonds, hydrophobic interactions, and π–π stacking. In contrast, TC maintained stable binding primarily through hydrogen bonds and hydrophobic forces, without the contribution of π–π stacking. Collectively, these structural insights imply that diverse JH components, whether through complex stacking or direct hydrogen bonding, possess the specific capacity to target TPH1, providing a molecular basis for the regulation of 5-HT biosynthesis.

### 3.7. Molecular dynamics (MD) simulations and binding free energy calculations

To computationally assess the stability and dynamic behavior of the ligand–protein complexes under physiological conditions, 100 ns MD simulations were performed for TPH1 complexed with TC, Theaflavins (TF, TF-3-G, TF-3′-G, TF-D-G), and the reference drug Fluoxetine (FLX).

**Fig. 7:**
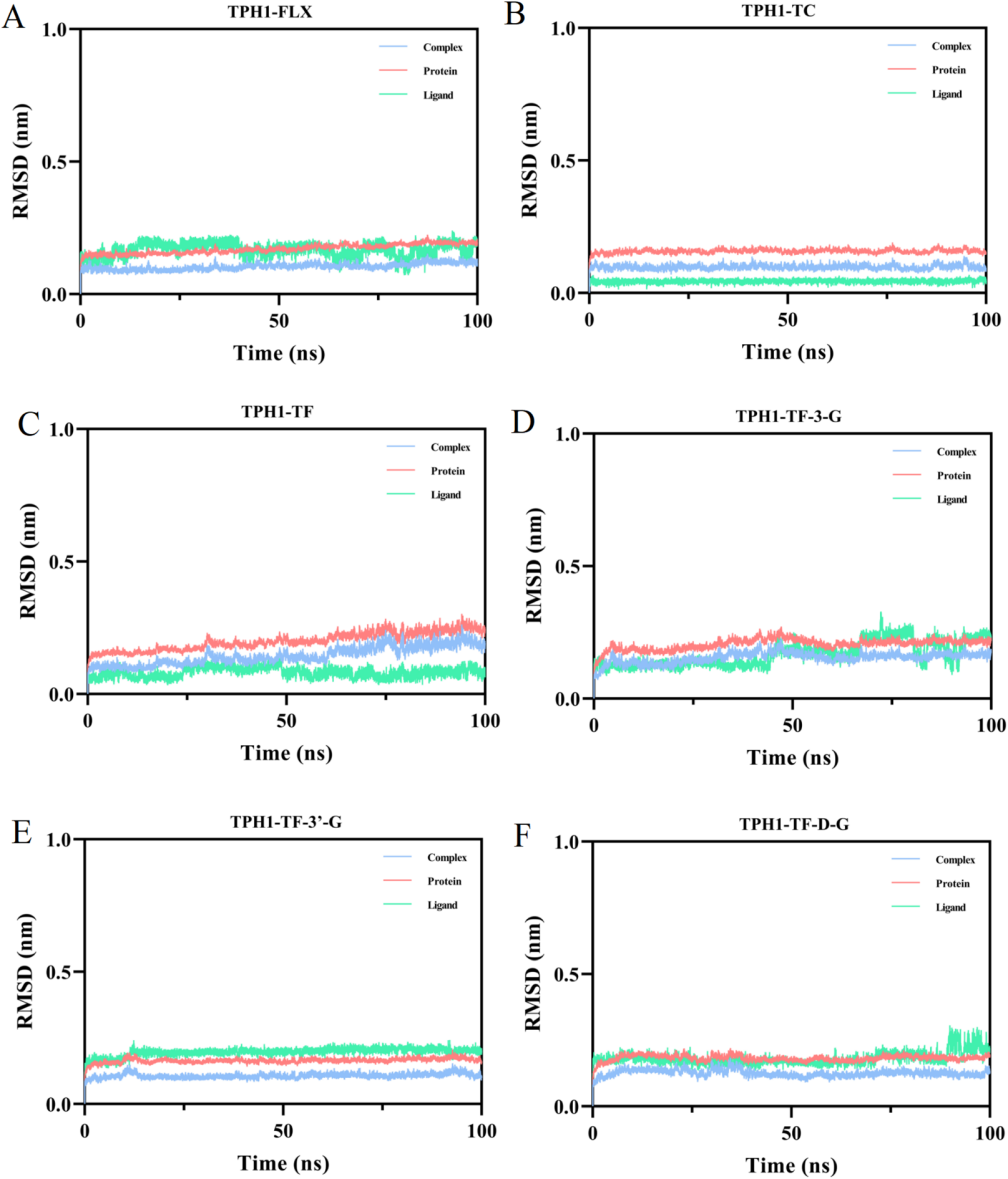
RMSD of the TPH1 protein backbone in complex with different ligands during 100 ns molecular dynamics (MD) simulations using GROMACS. (A–F) Trajectories for TPH1 complexed with FLX, TC, TF, TF-3-G, TF-3′-G, and TF-D-G, respectively.

System stability was first comprehensively evaluated. RMSD analysis (Fig. 7) indicated that all systems reached dynamic equilibrium rapidly. The TPH1-TC complex exhibited exceptional stability (RMSD: 0.098 ± 0.009 nm), comparable to the FLX reference, implying that the small-molecule alkaloid fits rigidly within the binding site. Similarly, the larger TPH1-TF-3′-G complex maintained a stable trajectory. Consistent with this, RMSF analysis (Fig. S3) confirmed a “locking” effect, where ligand binding significantly reduced residue flexibility in the TPH1's active pocket (<0.3 nm). Furthermore, global structural integrity was corroborated by *R*_g_ and SASA analyses (Fig. S4 and S5), which showed steady values throughout the simulation, confirming that the protein of TPH1 maintained its compactness. Free energy landscapes (FEL) (Fig. S6) further visualized this stability, revealing distinct low-energy basins that indicate the complexes (TPH1-TC, TPH1-Theaflavins and TPH1-FLX) settled into stable metastable conformations.

To quantify the energetic driving forces, hydrogen bond analysis and MM-PBSA calculations were performed. The number of hydrogen bonds formed in all complexes (TPH1-FLX, TPH1-TC, and TPH1-Theaflavins) remained steady, exhibiting only slight fluctuations (Fig. S7). Among these, TF-3′-G maintained a robust hydrogen bond network (mean: 4.67 ± 1.09), indicating persistent polar interactions. Crucially, binding free energy calculations (Table 3 and Fig. S8). Revealed that TF-3′-G possessed the strongest binding affinity (Δ*G*_binding_ = −41.36 kcal mol^−1^), followed closely by TF-D-G (−35.70 kcal mol^−1^); both significantly surpassed the positive control FLX (−27.49 kcal mol^−1^). Per-residue energy decomposition (Fig. S9) identified TPH1's amino acid residues (GLU317, TYR235, LEU236, PRO238, and GLN306), which contributed significantly (Δ*G*_res_ ≤ −2 kcal mol^−1^) through cooperative hydrogen bonds, hydrophobic interactions, and π–π stacking. Collectively, these atomistic simulations provide supporting computational evidence that theacrine and theaflavins, as key JH components, form stable, high-affinity interactions with TPH1. This interaction offers a mechanistic basis for the observed restoration of serotonin synthesis.

**Table 3:** Binding free energies and energy components of JH active compounds with TPH1 calculated by the MM-PBSA method

Energy component (kcal mol^−1^)	TPH1-FLX	TPH1-TC	TPH1-TF	TPH1-TF-3-G	TPH1-TF-3′-G	TPH1-TF-D-G
△VDWAALS	−29.36	−33.65	−41.13	−42.39	−32.68	−45.15
△EEL	−189.42	−24.32	−152.58	51.06	−193.44	8.57
△EPB	194.90	44.42	170.81	−25.15	190.13	6.09
△ENPOLAR	−3.62	−2.85	−4.96	−4.85	−5.37	−5.21
△EDISPER	0.00	0.00	0.00	0.00	0.00	0.00
△GGAS	−218.78	−57.98	−193.71	8.67	−226.12	−36.57
△GSOLV	191.28	41.57	165.85	−30.00	184.76	0.88
△TOTAL	−27.49	−16.40	−27.86	−21.33	−41.36	−35.70

## 4. Discussion

Our previous study demonstrated that JH mitigates CUMS-induced depression-like behaviors, potentially by modulating gut microbiota composition, brain neurochemicals, and cytokines.^14^ Building upon these findings, the current study employed serum metabolomics and computational biology to further decipher the potential therapeutic mechanisms. Here, we reveal that JH intervention induces a coordinated normalization of the serum metabolic profile, effectively reversing CUMS-induced perturbations. Notably, our analysis identified glycerophospholipid metabolism, arachidonic acid metabolism, and tryptophan metabolism as the critical pathways regulated by JH. These findings suggest that the metabolic correction is not driven by isolated markers but by a systemic restoration of key networks, which collectively modulate membrane lipid homeostasis, inflammatory signaling, and neurotransmitter precursor availability.

Metabolic stress in depression is often characterized by disrupted phospholipid homeostasis. We observed concerted changes in PC, LysoPC, and PE in the CUMS group (Fig. 2A), which were reversed by JH. Mechanistically, these shifts reflect not only altered phospholipid turnover but also the reorganization of membrane microdomains, which critically regulate receptor signaling efficiency.^21^ Furthermore, membrane phospholipids serve as the primary reservoir for arachidonic acid (AA).^22^ The CUMS-induced remodeling observed here likely acts as an upstream “substrate gate,” releasing AA to generate downstream pro-inflammatory lipid mediators.^23^ Consistent with this, we found elevated levels of AA-derived oxylipins, such as 12(*S*)-HETE, in the model group (Fig. 2A). By downregulating these lipid mediators, JH likely stabilizes cell membranes and blocks the supply of inflammatory signals, thereby severing the biochemical link between peripheral lipid remodeling and central neuroinflammation.^24,25^

Beyond lipid remodeling, the most critical metabolic recovery induced by JH was the regulation of the tryptophan metabolism axis. Under stress, tryptophan flux was diverted towards the kynurenine pathway, evidenced by reduced tryptophan and accumulated 3-hydroxyanthranilate (3-HAA). This shift is typically driven by inflammation-inducible enzymes (IDO1/TDO2), which deplete the substrate for 5-HT biosynthesis while generating downstream kynurenine catabolites.^26^ Crucially, the accumulation of 3-HAA indicates a pathological shift toward the kynurenine branch of the pathway.^27,28^ JH treatment successfully upregulated tryptophan and downregulated 3-HAA (Table 1 and Fig. 2A), which implies a potential amelioration of this pathological shunt. By dampening inflammation, JH reduces the diversion of tryptophan to the kynurenine pathway, creating a favorable metabolic environment for 5-HT restoration.^2,29^

To decode the molecular drivers linking these metabolic phenotypes to therapeutic targets, we integrated metabolomics with network pharmacology, pinpointing TPH1 as the critical bridge. TPH1 governs peripheral 5-HT synthesis, which dictates the systemic availability of tryptophan and influences central signaling *via* the gut–brain axis.^30–32^ Interestingly, our initial network analysis showed higher degree values for catechins, theanine and theaflavins compared to theacrine (TC) (Table S3). This discrepancy is likely attributable to a literature/database bias: these compounds have been extensively studied for decades, leading to abundant target annotations, whereas theacrine is a relatively novel pharmacological agent with fewer records. However, relying solely on topology can be misleading. Despite its lower network ranking, our subsequent molecular dynamics (MD) simulations demonstrated that theacrine exhibits exceptional binding stability with TPH1 (Fig. 7).

Our atomistic simulations further provided novel insights into a synergistic binding mechanism unique to Jianghua Kucha black tea, which contains both abundant theaflavins and theacrine. Specifically, theaflavin derivatives act as high-affinity “anchors,” achieving remarkable binding energies (*e.g.*, −41.36 kcal mol^−1^ for TF-3′-G) that significantly outperform the reference drug Fluoxetine, suggesting they can tightly lock onto the TPH1 catalytic pocket. Complementarily, theacrine acts as a “stabilizer.” While its binding energy is moderate, its RMSD trajectory was exceptionally smooth and low (0.098 ± 0.009 nm), indicating the formation of a rigid, highly stable complex with minimal fluctuation. This molecular synergy, where theaflavins provide potent affinity and theacrine provides structural stability, may contribute to the observed efficacy of JH in modulating the serotonergic system compared to single-compound interventions.

While the computational evidence for binding is strong, a potential paradox arises regarding the pharmacokinetics of theaflavins: despite their potent affinity for TPH1 *in silico* (Tables 2 and 3), these large molecules of catechin oxidation polymers typically exhibit low systemic bioavailability and poor intestinal absorption.^33^ However, this pharmacokinetic “limitation” may actually constitute a therapeutic advantage in the context of TPH1 regulation. Biologically, TPH1 is predominantly expressed in the enterochromaffin cells (ECs) of the intestinal epithelium, which are responsible for synthesizing over 90% of the body's peripheral 5-HT.^34^ The low absorption rate of theaflavins results in their accumulation at high concentrations within the intestinal lumen, facilitating direct physical contact with the mucosal lining where ECs reside. Therefore, we postulate that theaflavins may not require systemic circulation entry to exert their effects; instead, they likely target intestinal TPH1 *in situ*. This local modulation of peripheral 5-HT synthesis can subsequently influence central nervous system function *via* the gut–brain axis, particularly through vagus nerve signaling.^35^

Despite these promising findings, several limitations of the present study should be acknowledged. While the integration of serum metabolomics and rigorous molecular dynamics simulations provides a compelling multi-scale structural and metabolic framework, direct biological experimental validation is lacking. The exact regulation of TPH1 protein expression, its enzymatic activity *in vivo*, and the specific suppression of kynurenine pathway rate-limiting enzymes (*e.g.*, IDO1 or TDO2) require further empirical evidence. Future studies employing *in vitro* enzymatic activity assays, western blotting, and specific TPH1 knockout animal models are warranted to biologically validate the computational targets and metabolic shunting mechanisms proposed herein.

## 5. Conclusions

In summary, this study integrates serum metabolomics, network pharmacology, and molecular dynamics to elucidate the antidepressant mechanism of Jianghua Kucha black tea (JH). We observed that JH significantly reprograms peripheral metabolism in CUMS mice, primarily by stabilizing membrane lipid homeostasis through the modulation of glycerophospholipids and arachidonic acid, which in turn dampens inflammation and potentially rectifies the pathological tryptophan–kynurenine shunt. Furthermore, multi-scale computational analyses predicted TPH1 as a potential pivotal molecular target linking these metabolic phenotypes to tea bioactives. Specifically, MD simulations suggested that theacrine and theaflavins could potentially form stable, high-affinity interactions with TPH1, providing a potential structural basis for the observed metabolic regulation. Collectively, these findings offer promising evidence for developing JH as a functional food for depression management and propose a predictive mechanistic framework, paving the way for future *in vivo* functional validation of these specific targets.

## Author contributions

Xiaolu Yang: writing – review and editing, writing – original draft, methodology, investigation, formal analysis, data curation. Guifen Wang: conceptualization, methodology, formal analysis, investigation, writing – original draft. Chuanwei Zhen: formal analysis, investigation. Zhongjun Yan: formal analysis. Wei Liu: investigation. Aixiang Hou: conceptualization, formal analysis, writing – review and editing, funding acquisition. Wenliang Wu: conceptualization, formal analysis, writing – review and editing, project administration, funding acquisition.

## Conflicts of interest

The authors declare that they have no known competing financial interests or personal relationships that could have appeared to influence the work reported in this paper.

## Supplementary Material

RA-016-D6RA00952B-s001

RA-016-D6RA00952B-s002

## Data Availability

The datasets used and/or analyzed during the current study are available from the corresponding author on reasonable request. All data generated or analyzed during this study are included in this published article (and its supplementary information (SI) files). Supplementary information is available. See DOI: https://doi.org/10.1039/d6ra00952b.
