# Supplementary material for: Theacrine-rich Jianghua Kucha black tea alleviates depression via remodeling systemic tryptophan metabolism and targeting TPH1: insights from metabolomics and molecular simulation
Source: RSC Adv. 2026 Jul 2;16(34):33170–83. doi: 10.1039/d6ra00952b (PMC13326656; doi:10.1039/d6ra00952b)
Supplement: RA-016-D6RA00952B-s001 [file RA-016-D6RA00952B-s001.pdf]

Supplementary Information (SI) for RSC Advances.
This journal is © The Royal Society of Chemistry 2026

Theacrine-rich Jianghua Kucha Black Tea Alleviates Depression via Remodeling Systemic Tryptophan Metabolism and Targeting TPH1: Insights from Metabolomics and Molecular Simulation

Xiaolu Yang [a, b, #], Guifen Wang [a, b, #], Chuanwei Zheng [a], Zhongjun Yan [b], Wei Liu [d], Aixiang Hou [a, *], Wenliang Wu [b, c, **]

[a] College of Food Science and Technology, Hunan Agricultural University, Changsha, Hunan 410128, China

[b] Hunan Institute of Tea Research, Hunan Academy of Agricultural Sciences, Changsha, Hunan 410125, China

[c] Longping Agricultural College, Hunan University, Changsha, Hunan 410082, China

[d] Chenzhou Institute of Agricultural Sciences, Chenzhou, Hunan 423000, China

# These authors contributed equally to this work

* Corresponding author: Email: aixianghou@163.com

** Corresponding author: Email: wwlvip8@163.com

Supplementary material

Table S1 Results from pathway analysis

Metabolic pathways	Total	Expected	Hits	Raw p	-log10 (p)	Holm adjust	FDR	Impact
Glycerophospholipid metabolism	36	0.3392	4	0.0002522 2	3.5982	0.020178	0.020178	0.24277
Arachidonic acid metabolism	44	0.41457	3	0.007109	2.1482	0.56161	0.28436	0.27659
Glycine, serine and threonine metabolism	33	0.31093	2	0.036975	1.4321	1	0.8798	0
Linoleic acid metabolism	5	0.047111	1	0.046288	1.3345	1	0.8798	0
Tryptophan metabolism	41	0.38631	2	0.048988	1.3099	1	0.8798	0.19462
Valine, leucine and isoleucine biosynthesis	8	0.075377	1	0.073093	1.1361	1	0.97457	0
alpha-Linolenic acid metabolism	13	0.12249	1	0.11621	0.93475	1	1	0
Citrate cycle (TCA cycle)	20	0.18844	1	0.17344	0.76086	1	1	0.04412
Pyruvate metabolism	23	0.21671	1	0.19689	0.70578	1	1	0.0283
One carbon pool by folate	26	0.24497	1	0.21972	0.65814	1	1	0
Glutathione metabolism	28	0.26382	1	0.2346	0.62967	1	1	0.00709
Glyoxylate and dicarboxylate metabolism	32	0.30151	1	0.26357	0.57911	1	1	0
Glycosylphosphatidylinositol (GPI)-anchor biosynthesis	32	0.30151	1	0.26357	0.57911	1	1	0.03665
Biosynthesis of unsaturated fatty acids	36	0.3392	1	0.29151	0.53534	1	1	0
Arginine and proline metabolism	36	0.3392	1	0.29151	0.53534	1	1	0.02442
Drug metabolism - other enzymes	39	0.36746	1	0.31182	0.5061	1	1	0
Valine, leucine and isoleucine degradation	40	0.37688	1	0.31847	0.49694	1	1	0
Primary bile acid biosynthesis	46	0.43342	1	0.35711	0.4472	1	1	0
Purine metabolism	70	0.65955	1	0.49214	0.30792	1	1	0.01146

Table S2 The main biochemical components of JH. Amino acids (AAs); Tea polyphenols (TPs); Soluble sugars (SS); Gallocatechin (GC); Epicatechin (EGC); Catechin (C); Epicatechin (EC); Epigallocatechin gallate (EGCG); Gallocatechin gallate (GCG); Epicatechin gallate (ECG); Theobromine (TB); Theophylline (THEO); Caffeine (CAF); Theacrine (TC); Theaflavins (TFs); Thearubigins (TRs)

Components	JH（%）
AAs	8.69±0.17
TPs	29.77±1.6
SS	6.28±0.49
GC	0.39±0.05
EGC	0.45±0.06
C	0.74±0.10
EC	0.34±0.04
EGCG	0.96±0.03
GCG	0.18±0.02
ECG	1.48±0.02
Total catechins	4.33±0.60
TB	0.23±0.06
THEO	0.14±0.00
CAF	3.75±0.67
TC	1.44±0.42
TFs	1.53±0.11
TRs	9.79±0.60

Note: Data for main biochemical components of JH were retrieved from our previous study[14]. doi: https://doi.org/10.1016/j.foodres.2024.115306

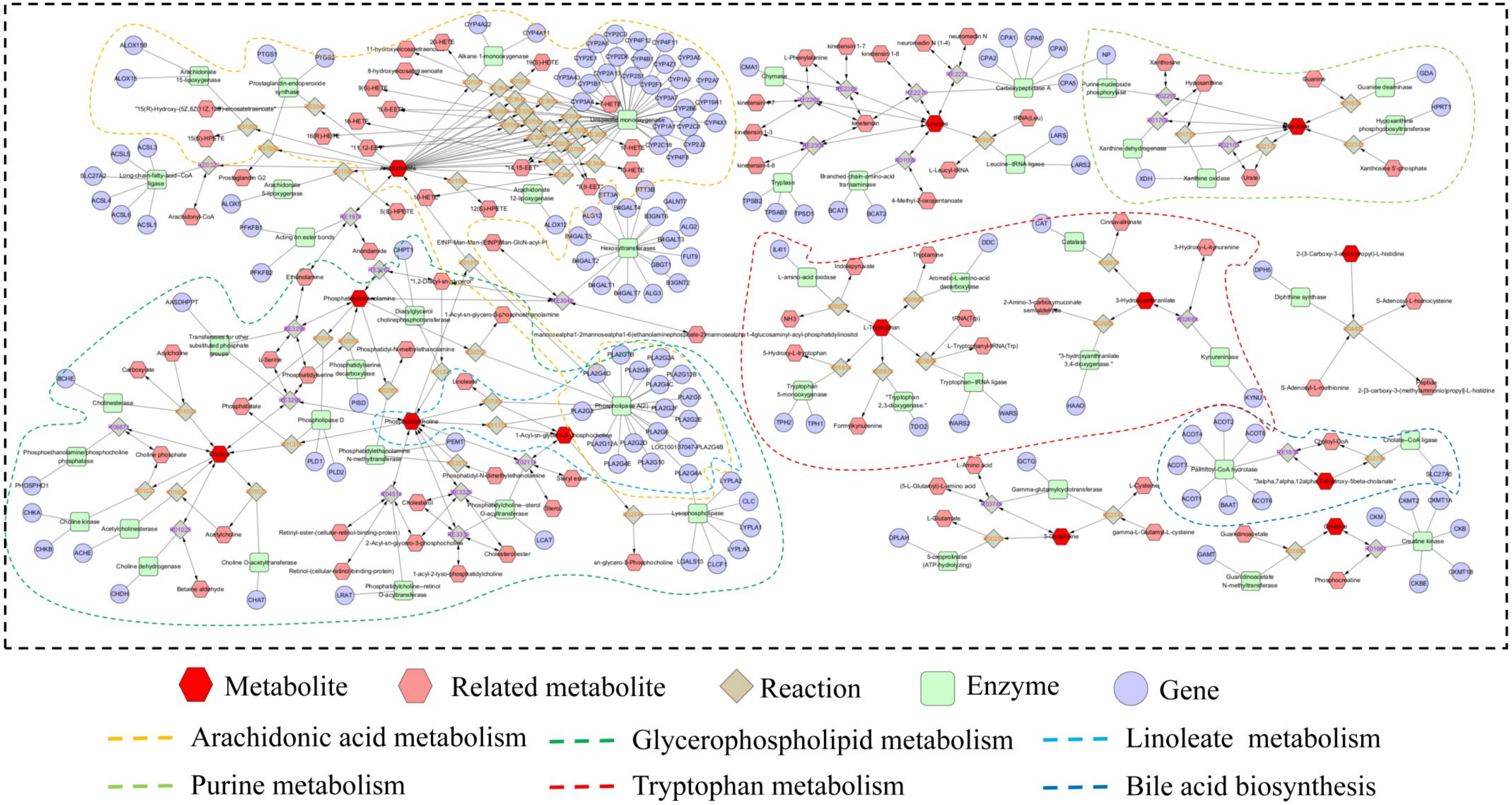

Figure S1: Construction of the integrated "Metabolite-Reaction-Enzyme-Target" interaction network.

A.

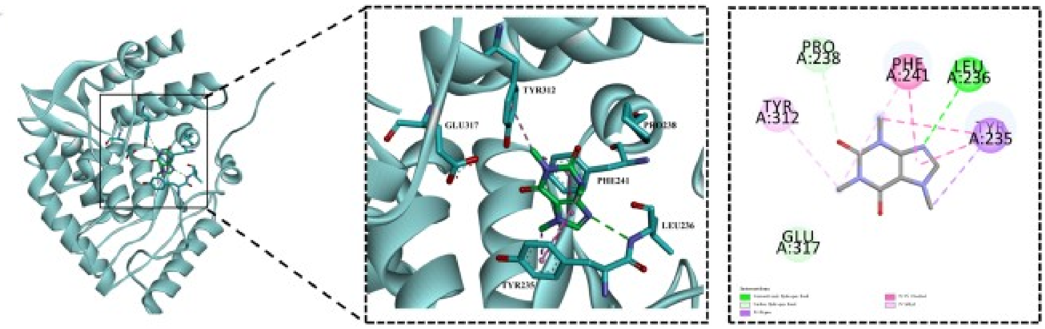
TYR312
GLU317
PHE238
PHE241
LEU236
TYR235
PRO A:238
PHE A:241
LEU A:236
TYR A:312
TYR A:235
GLU A:317

B.

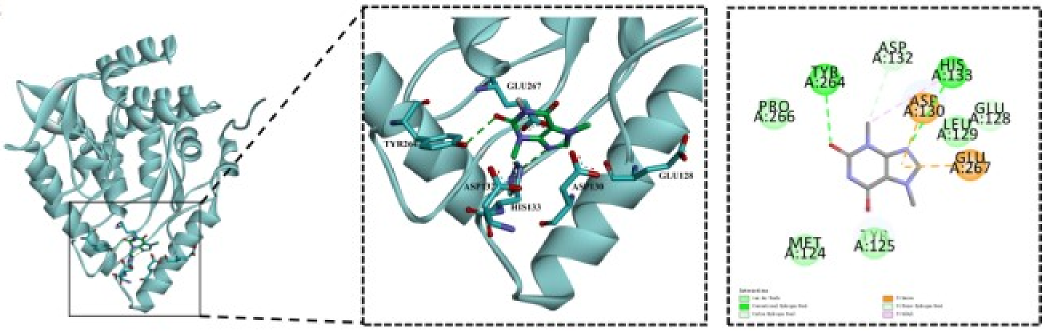
GLU267
TYR264
ASP132
ASP130
GLU128
HIS133
ASP A:132
TYR A:264
HIS A:133
PRO A:266
ASP A:130
GLU A:128
LEU A:129
GLU A:267
MET A:124
TYR A:125

C.

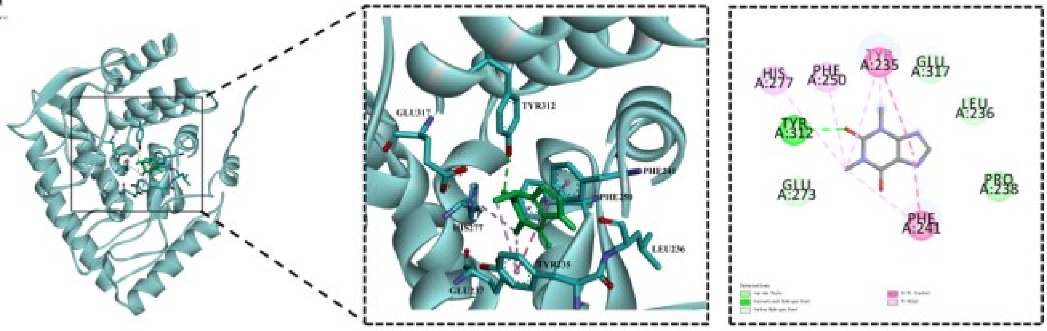
GLU317
TYR312
PHE241
PHE250
HIS277
LEU236
TYR235
GLU273
TYR A:235
GLU A:317
HIS A:277
PHE A:250
LEU A:236
TYR A:312
GLU A:273
PRO A:238
PHE A:241

D.

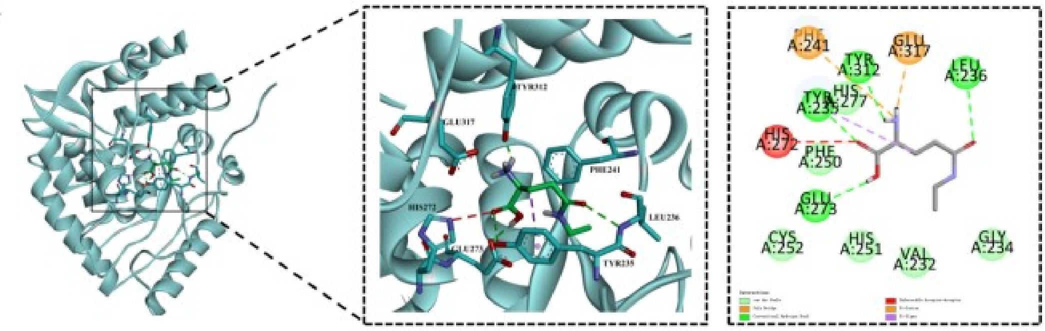
TYR312
GLU317
PHE241
HIS272
GLU273
LEU236
TYR235
PHE A:241
TYR A:312
GLU A:317
LEU A:236
TYR A:235
HIS A:277
HIS A:272
PHE A:250
GLU A:273
CYS A:252
HIS A:251
VAL A:232
GLY A:234

E

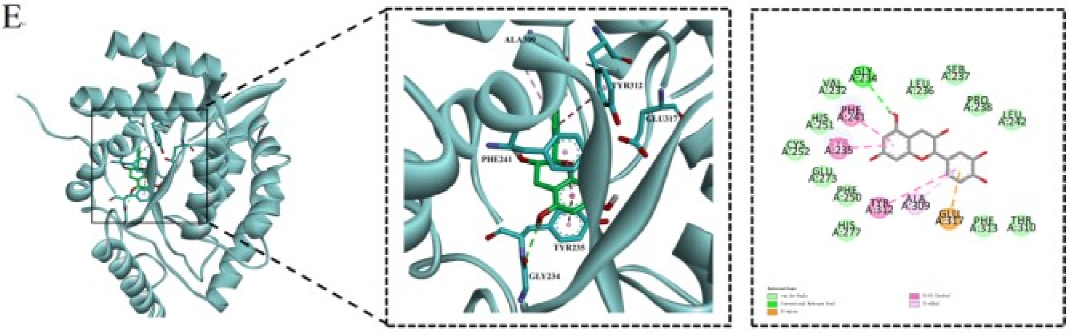
ALA309
TYR312
GLU317
PHE241
TYR235
GLY234
VAL A:232
GLY A:234
LEU A:236
SER A:237
PRO A:238
LEU A:242
HIS A:251
PHE A:241
CYS A:252
A:235
GLU A:273
PHE A:250
TYR A:312
ALA A:309
GLU A:317
PHE A:313
THR A:310
HIS A:277

F

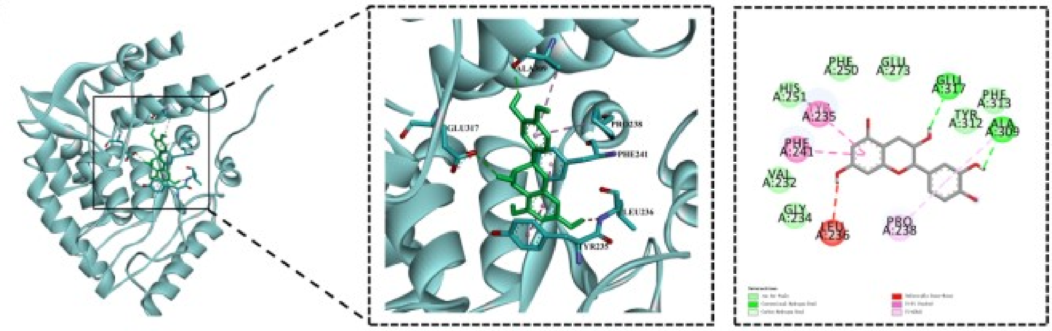
GLU317
PRO238
PHE241
LEU236
TYR235
PHE A:250
GLU A:273
GLU A:317
HIS A:251
A:235
PHE A:313
TYR A:312
ALA A:309
PHE A:241
VAL A:232
GLY A:234
LEU A:236
PRO A:238

G

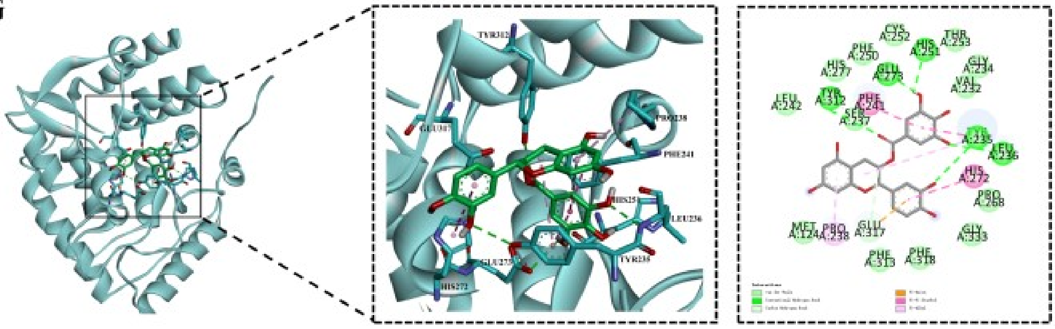
TYR312
GLU317
PRO238
PHE241
HIS251
LEU236
TYR235
GLU273
HIS272
CYS A:252
THR A:253
PHE A:250
HIS A:251
HIS A:277
GLU A:273
GLY A:234
VAL A:232
LEU A:242
TYR A:312
PHE A:241
SER A:237
A:235
LEU A:236
HIS A:272
PRO A:268
MET A:124
PRO A:238
GLU A:317
GLY A:333
PHE A:313
PHE A:318

H

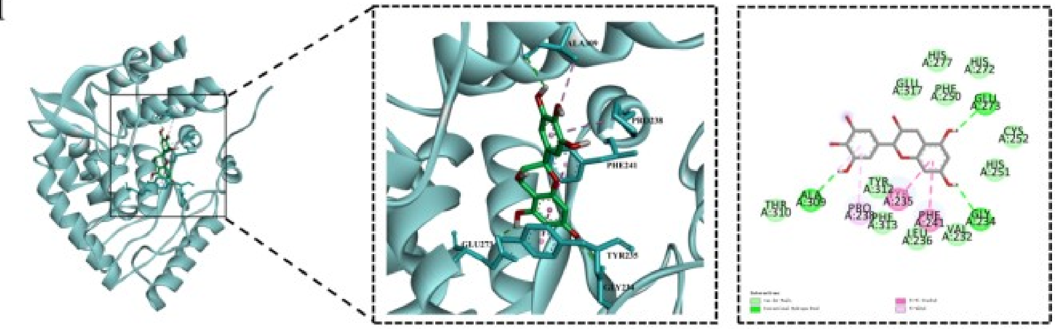
ALA309
PRO238
PHE241
GLU273
TYR235
GLY234
HIS A:277
HIS A:272
GLU A:317
PHE A:250
GLU A:273
CYS A:252
HIS A:251
THR A:310
ALA A:309
TYR A:312
A:235
PRO A:238
PHE A:313
PHE A:241
LEU A:236
VAL A:232
GLY A:234

I

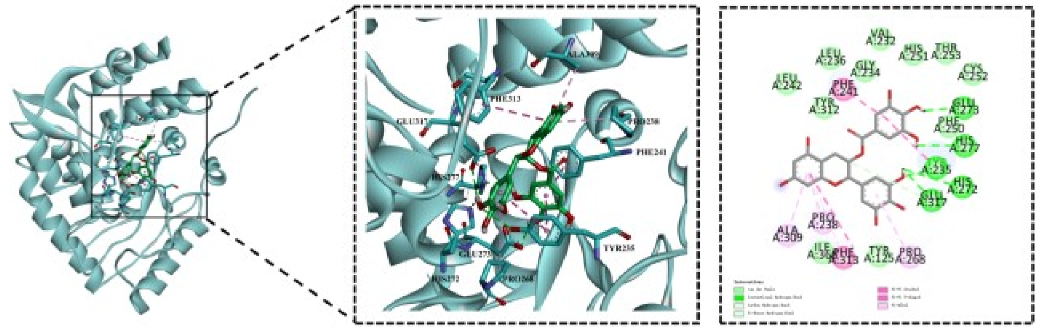

J

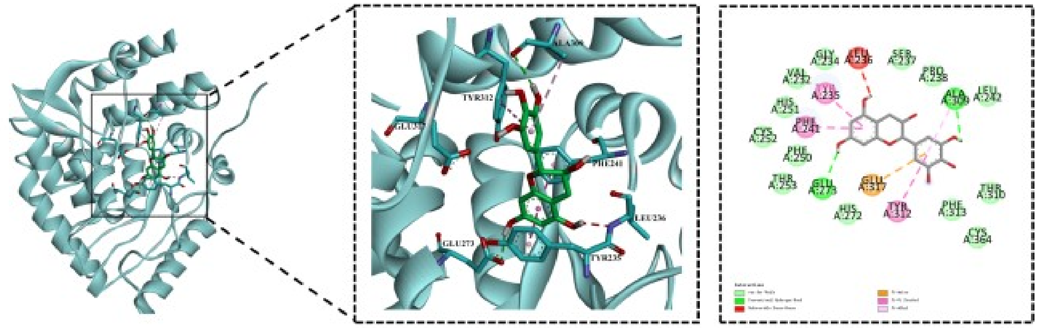

K

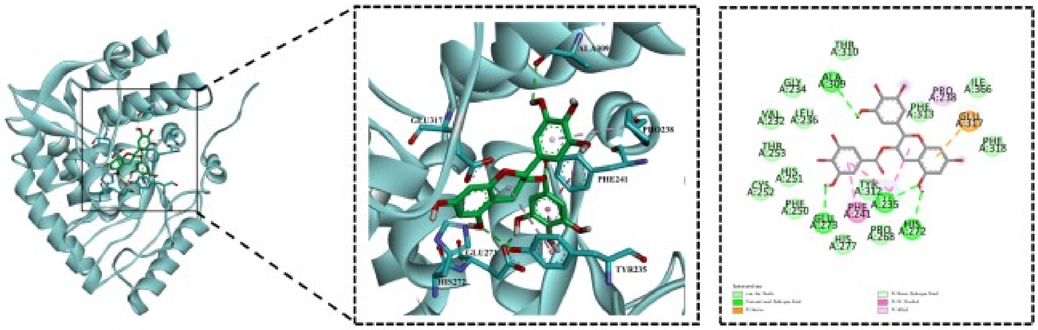

Figure S2: Molecular docking poses of other JH components with TPH1. The 2D and 3D binding modes are shown for: (A) CAF (-6.6 kcal/mol); (B) TB (-6.1 kcal/mol); (C) THEO (-6.3 kcal/mol); (D) TA (-6.2 kcal/mol); (E) C (-10.1 kcal/mol); (F) EC (-9.5 kcal/mol); (G) ECG (-11.1 kcal/mol); (H) EGC (-9.4 kcal/mol); (I) EGCG (-10.5 kcal/mol); (J) GC (-10 kcal/mol); (K) GCG (-10.5 kcal/mol).

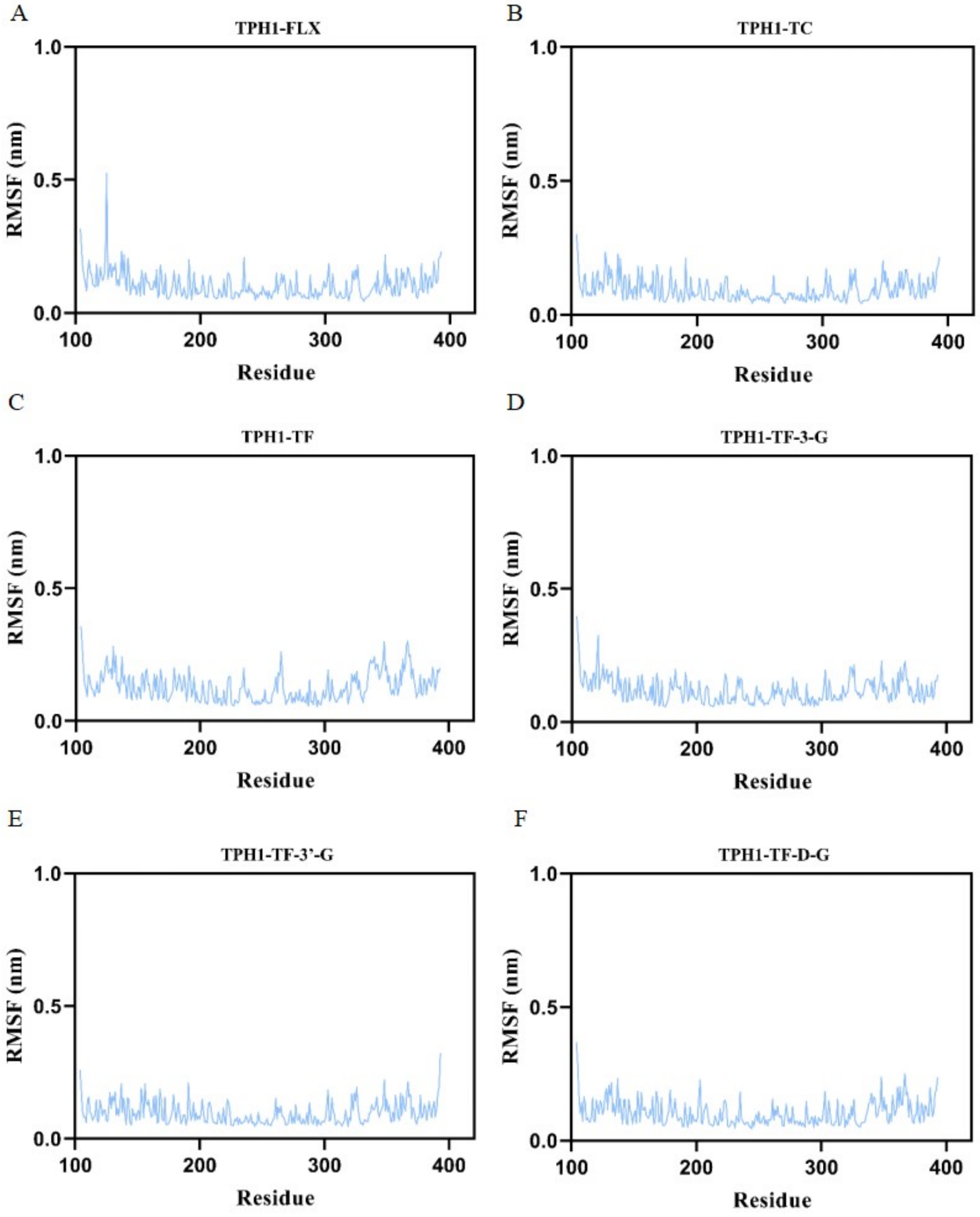

Figure S3: Root Mean Square Fluctuation (RMSF) analysis of Protein-Ligand Complexes in MD Simulations. (A) TPH1-FLX, (B) TPH1-TC, (C) TPH1-TF, (D) TPH1-TF-3-G, (E) TPH1-TF-3’-G, and (F) TPH1-TF-D-G.

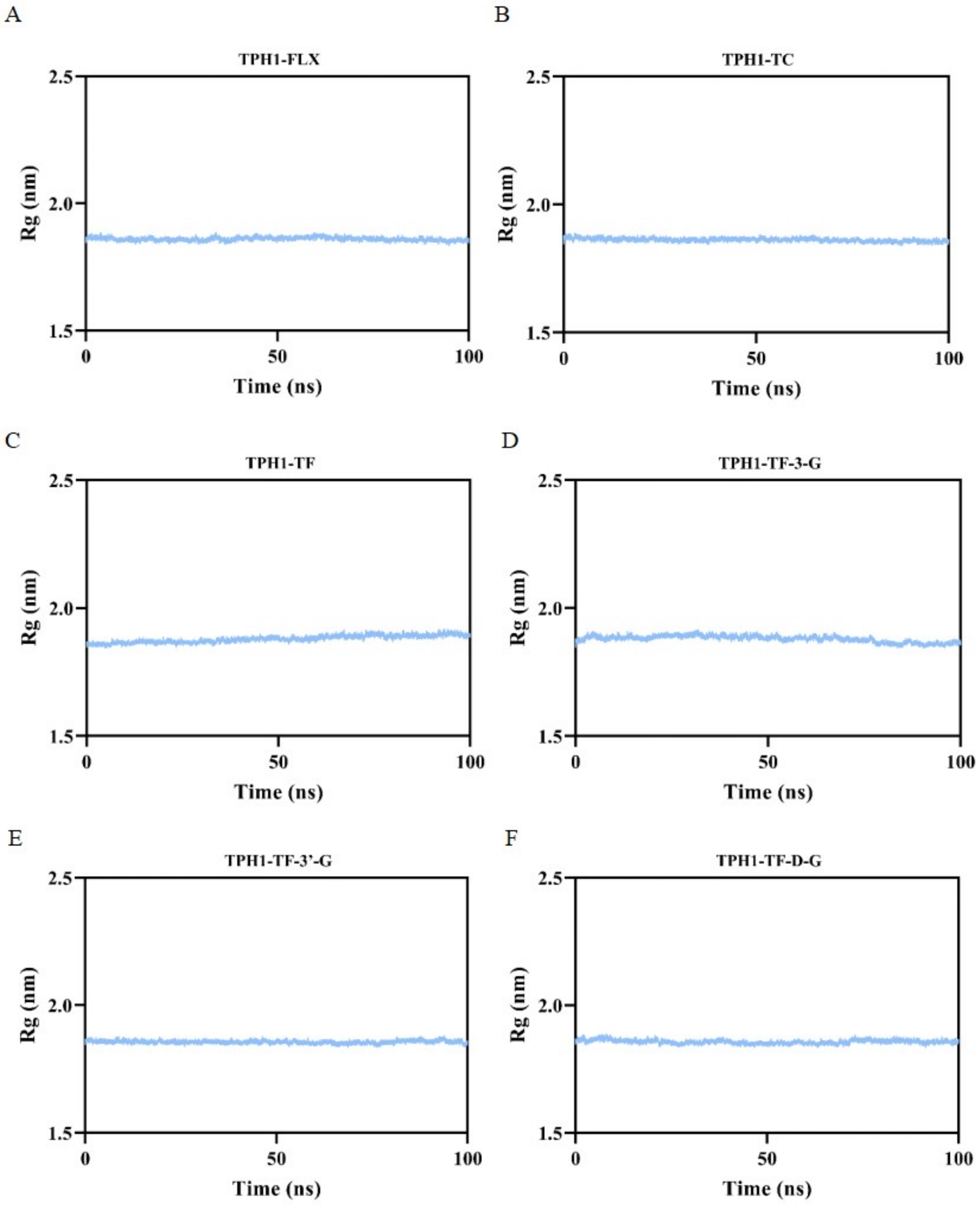

Figure S4: Radius of Gyration (Rg) of the protein-ligand complexes during 100 ns simulation. (A) TPH1-FLX, (B) TPH1-TC, (C) TPH1-TF, (D) TPH1-TF-3-G, (E) TPH1-TF-3'-G, and (F) TPH1-TF-D-G.

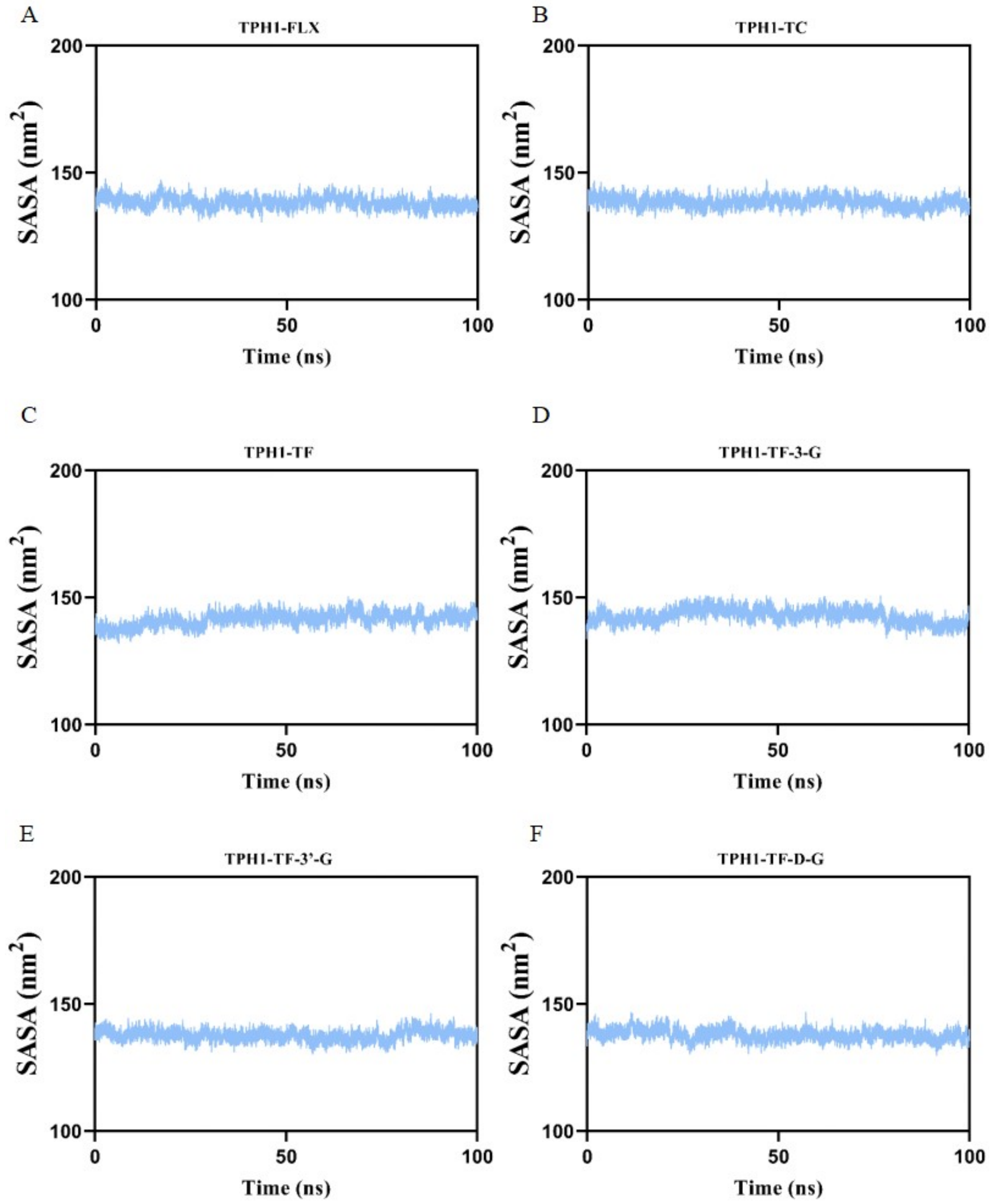

Figure S5: Solvent Accessible Surface Area (SASA) of the protein-ligand complexes during 100 ns simulation. (A) TPH1-FLX, (B) TPH1-TC, (C) TPH1-TF, (D) TPH1-TF-3-G, (E) TPH1-TF-3'-G, and (F) TPH1-TF-D-G.

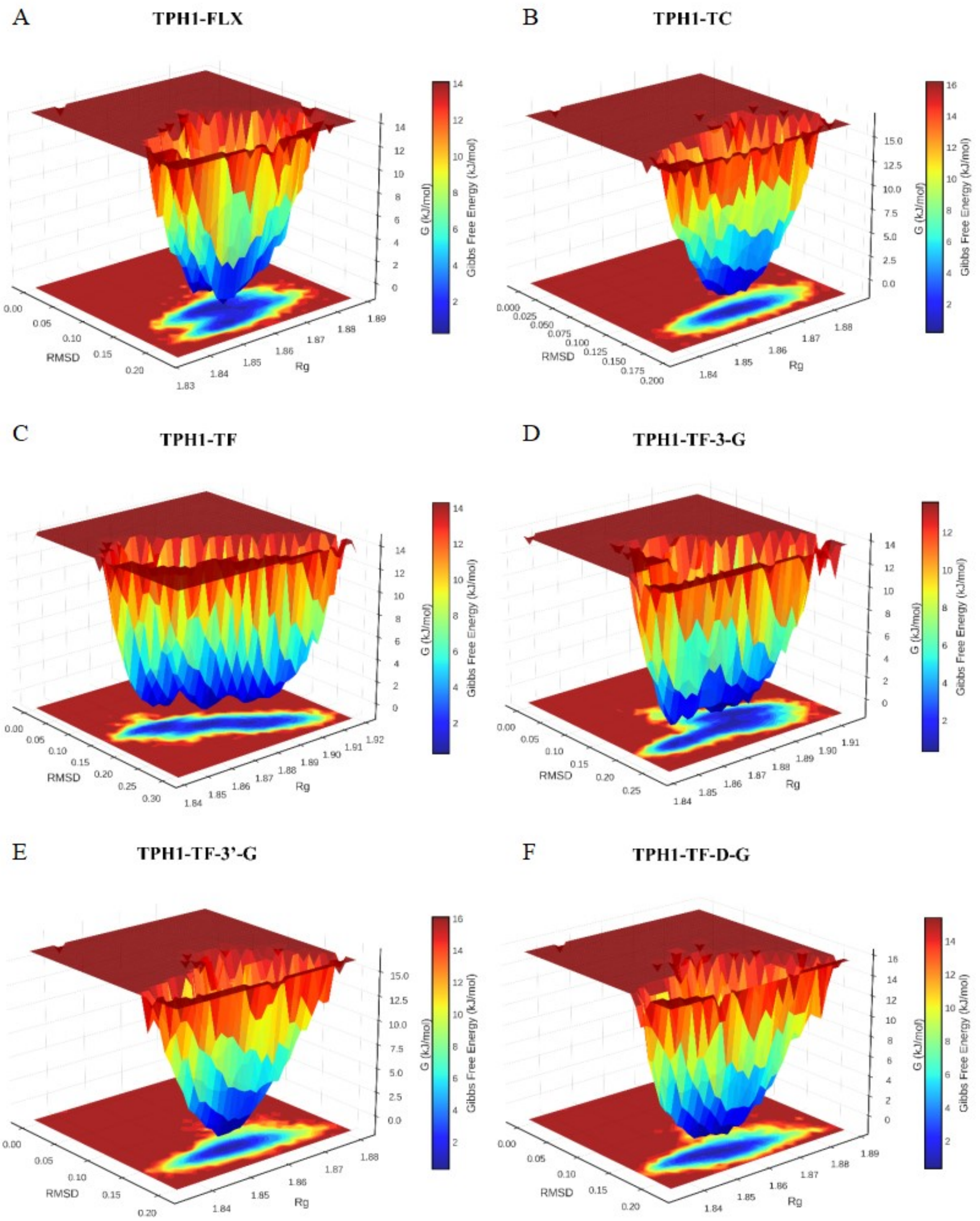

Figure S6: Free Energy Landscape (FEL) of the Protein-Ligand Complexes. (A) TPH1-FLX, (B) TPH1-TC, (C) TPH1-TF, (D) TPH1-TF-3-G, (E) TPH1-TF-3’-G, and (F) TPH1-TF-D-G.

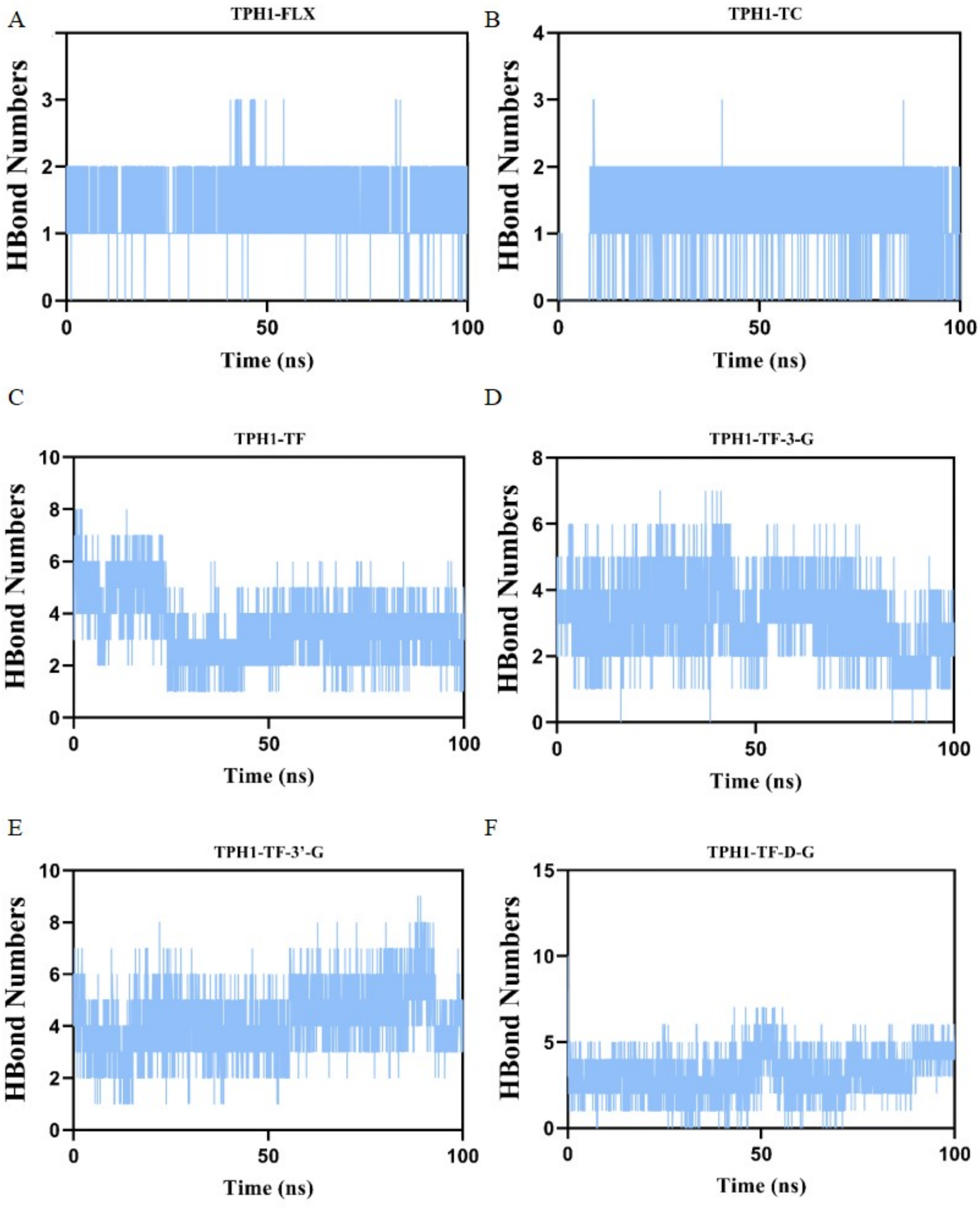

Figure S7: Hydrogen bond analysis of the Protein-Ligand Complexes. (A) TPH1-FLX, (B) TPH1-TC, (C) TPH1-TF, (D) TPH1-TF-3-G, (E) TPH1-TF-3'-G, and (F) TPH1-TF-D-G.

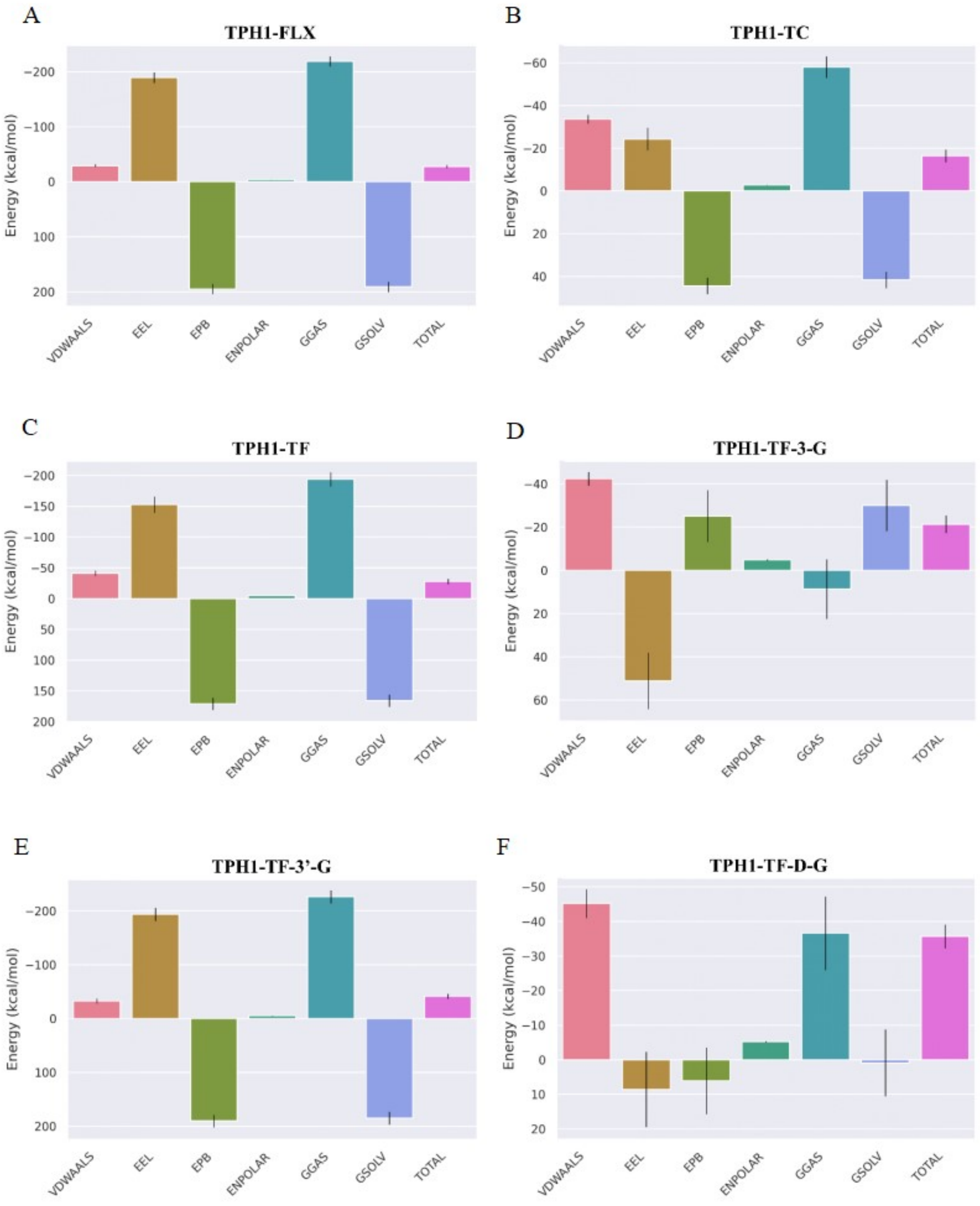

Figure S8: The total energy analysis of the protein–ligand complexes in MD. (A) TPH1-FLX, (B) TPH1-TC, (C) TPH1-TF, (D) TPH1-TF-3-G, (E) TPH1-TF-3’-G, and (F) TPH1-TF-D-G.

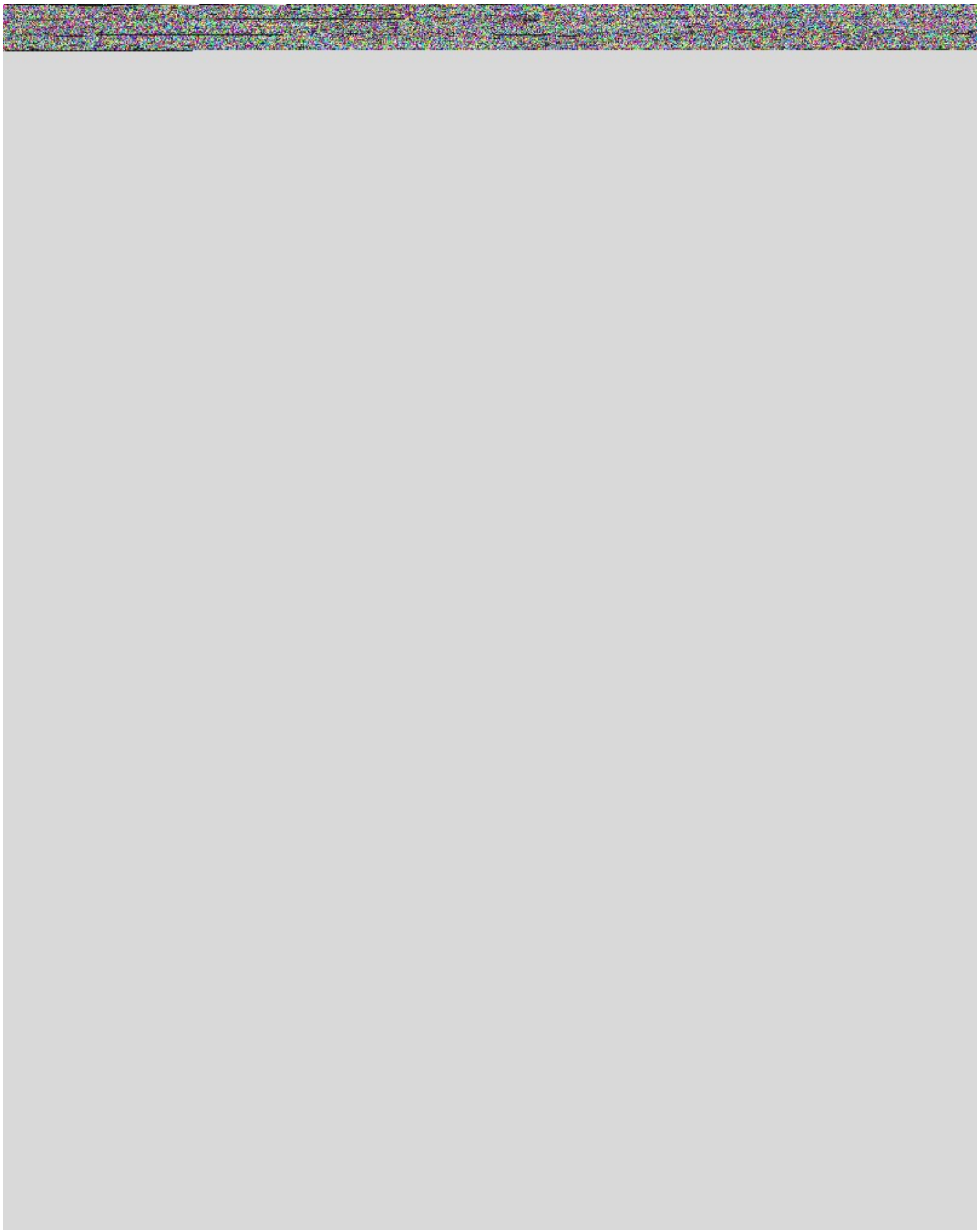

Figure S9: Per-residue binding free energy decomposition analysis (PB model). (A) TPH1-FLX, (B) TPH1-TC, (C) TPH1-TF, (D) TPH1-TF-3-G, (E) TPH1-TF-3'-G, and (F) TPH1-TF-D-G.
